# Both religious and secular ethics to achieve both happiness and health: Panel data results based on a dynamic theoretical model

**DOI:** 10.1371/journal.pone.0301905

**Published:** 2024-04-17

**Authors:** Fabio Zagonari

**Affiliations:** Dipartimento di Scienze per la Qualità della Vita, Università di Bologna, Rimini, Italy; The University of Adelaide - North Terrace Campus: The University of Adelaide, AUSTRALIA

## Abstract

This paper evaluates the direct and indirect impacts (and their interactions) of individual and social ethics from (primary, secondary, tertiary) education and religion (Buddhism, Christianity, Hinduism, Islam, Judaism) on health and happiness in alternative religious contexts (majority and minority religions) and for alternative education policies (gross enrolment and per-student expenditure). It also specifies the time lag for the short-run indirect impact (and its size) of happiness on health and the long-run equilibria of both happiness and health. The statistical results show that there is no religious or secular ethics with beneficial impacts on both happiness and health at both the individual and social levels. Next, education policies have similar impacts on both happiness and health in all religious contexts, while most religious ethics have larger beneficial impacts on health and happiness if coupled with social and individual education policies, respectively. Combined statistical and analytical results show that the largest short-run indirect impact of happiness on health occurs after 4 years, where 1 out of 10 points of happiness produces approximately 3 additional years of healthy life expectancy at birth. Next, the long-run equilibria of both happiness and health are globally stable and are achieved after 8 years through oscillation dynamics.

## Introduction

Education (EDU) and religion (REL) could provide individual-level knowledge and ethics about healthy behaviours (e.g., [1–4]). For example, religion and science suggest avoiding tobacco, alcohol and drug use; theology and philosophy provide meanings to help one cope with stress and depression. However, a decline in the share of religious people could favour a better environment for medical research due to the sceptical attitude of some religious individuals towards science.

EDU and REL could provide social capital and ethics about healthy behaviours (e.g., [5–15]). For example, religion provides formal and informal networks of emotional, social and material support, while science provides suggestions for preventing and healing social diseases with better practices and diagnostic techniques, as well as social norms and networks. However, an increase in the share of religious people could favour a decrease in public health expenditure due to the social services some individuals receive from their religious community.

EDU and REL provide individual-level knowledge and ethics about happy attitudes. For example, theology and philosophy provide meanings and purposes to help one deal with or adapt to adverse life events, as well as to buffer anxiety arising from fear of death; religion and culture help people better understand and exploit their personal potentials. However, religious beliefs in God and afterlife could make people find individual and social roles in spite of failures in efficiently exploiting their personal resources and skills (e.g., [16–22]).

EDU and REL provide social capital and ethics about happy attitudes. For example, institutional and social trust, social ties, sense of belonging, civic mindedness, social norms, and social networks could work to bring people together in communities at a national level, while religion could work to bring people together in communities at a level lower than the national level (e.g., [23–26]).

I will refer to 5 religions (i.e., Buddhism, BUD; Christianity, CHR; Hinduism, HIN; Islam, ISL; and Judaism, JUD) (using both percentages to depict individual ethics and majorities to depict social ethics) and 3 education levels (primary, P; secondary, S; and tertiary, T) (using both gross enrolment percentages as a proxy of social capital and per-student education expenditures as a proxy of individual knowledge). Note that the previous references are limited to the last 5 years of cross-country empirical papers involving more than 2 countries since the focus is on ethical differences.

Moreover, while health and happiness might be linked with a short-run time lag, they might also converge to a long-run equilibrium [27]. I will refer to life expectancy at birth (LEB) or healthy LEB (HLEB) for health and life satisfaction (LS) for happiness since the focus is on individuals’ lives.

Finally, given the prevailing religious and secular individual and social ethics, health also depends on health care systems (e.g., [28, 29]), whereas individual knowledge and social capital from EDU also depend on education quality systems (e.g., [30, 31]). I will refer to total health expenditure per capita and governmental education expenditure per capita as proxies of the effectiveness of health care and education systems, respectively, together with GDP per capita and the Gini index to differentiate more- or less-developed and unequal countries. Note that these four variables will eliminate the impacts of ethics on happiness and health from the consequences of alternative health care and education systems, as well as from different development and inequality degrees, since the focus is on the ethical differences in individuals’ lives.

The *purpose* of this paper is not only to evaluate the direct and indirect *impacts* (and their interactions) of individual and social ethics from EDU and REL on health and happiness in alternative religious contexts (i.e., majority and minority religions) and for alternative education policies (gross enrolment and per-student expenditure) but also to specify the time lag for the short-run indirect impacts (and its size) of happiness on health and the (globally stable) long-run equilibria of both happiness and health.

To do so, I refer to Zagonari [27] for the theoretical dynamic model. Moreover, I construct a balanced panel dataset for 162 countries from 2000 to 2020 by completing data from World Happiness Reports and World Bank Indicators using data from the World Health Organisation for HLEB and the World Value Survey for LS. Finally, I estimate a dynamic panel data two-equation system by using fixed effects 3-stage least squares (3SLS), where the fixed effects method controls for time-invariant omitted variables or unobserved characteristics that differ across countries (e.g., cultural peculiarities), by removing the effects of those characteristics and thus assessing the net effect of independent variables on the dependent variable.

## Methods

### Literature

The literature about the possible impacts of REL or EDU on happiness or health can be summarised in the following statement: REL is good for happiness, but it is bad for health, whereas EDU is good for health, but it is bad for happiness. However, the literature is affected by some methodological problems (i.e., the uncommon use of panel data with a sufficiently large number of observations per unit, as well as the rare use of HLEB instead of LEB) and by some unanswered questions (i.e., the potential impacts of the main 5 religions, namely, Buddhism, Christianity, Hinduism, Islam, and Judaism, together with the 3 main education levels, namely, primary, secondary and tertiary, on the possible dynamic interrelationships between happiness and health in the short and long run from a social and individual perspective).

Table 1 summarises *all* articles that are, to the best of my knowledge, based on panel data about any relationship between EDU or REL and happiness or health. Note that there are no articles on the dynamic interrelationships between happiness and health, although Pierewan & Tampubolon [32] find a reciprocal impact of happiness and health within a static framework.

**Table 1:** Summary of the empirical literature about the education or religion impacts on happiness or health based on panel data. Note that variables in brackets (i.e., (SRH) and (LEB)) are independent variables.

	HAP	HEA	N. countries	N. years	N. EDU	N. REL
Adegoke et al. (2022)		LEB	25 SSA	21	HCI	
Adeleye et al. (2022)		LEB	19 MENA	40	HCI	
Ahmadiani et al. (2022)	LS	(SRH)	78	6	3	
Bayati et al. (2013)		LEB	21 EM	13	HDI	
Hamidi et al. (2018)		LEB	18 MENA	15	years	
Hauck et al. (2016)		LEB	54 LIC	22	P	
Herzer (2022)		LEB	17 OECD	6	years	CHR
Islam (2020)		LEB	5 SA	5	ALR	
Nikolova & Popova (2021)	LS	(LEB)	91	6	S	
Perez & Rohde (2022)	SWB	SRH	4 OECD	15	T	3
Ray & Linden (2020)		LEB	195	10	P	
Schenkman & Bousquat (2021)		LEB	161	5	HDI	
Sharma (2018)		LEB	17 OECD		years	
This study	LS	LEB&HLEB	162	21	3	5

Abbreviations: HAP = happiness, LS = life satisfaction, SWB = subjective well-being, HEA = health, LEB = life expectancy at birth, SRH = self-reported health, SSA = Sub-Sahara Africa, MENA = Middle East and North Africa, EM = East Mediterranean, SA = South Asia, LIC = Low Income Countries, HCI = Human Capital Index, HDI = Human Development Index, ALR = Adult Literacy Rate, OECD = Organisation For Economic Cooperation And Development, P = primary, S = secondary, T = tertiary, CHR = Christianity.

In particular, the literature misses the interpretation of results in terms of individual and social ethics from EDU and REL. Moreover, Boháček et al. [33] and Rueda-Salazar et al. [34] use HLEB but do so in a cross-country analysis. Finally, there are no articles that combine the 5 main religions and the 3 main education levels, apart from Letelier and Saez-Lozano [18] in a cross-country framework.

This study combines and develops the extant research. In particular, I use data on observed statuses (i.e., HLEB, LEB based on averages of *recorded* data at a country level) rather than self-reported statuses (i.e., averages of answers obtained by surveys at an individual level where respondents are asked to rate their *perception* about their status of health in the last one year using scales usually based on 3 or 5 steps): thus, individual knowledge or social capital will have impacts on happiness and health only if these factors are translated into individual and social ethics (e.g., you think that you should be grateful to God as a believer or appreciate nature as an unbeliever, but you are unsatisfied with your life; you know that smoking is bad for your health and goes against a religious precept, but your demanding job seems to require it).

Moreover, considering both religious and secular ethics (i.e., 5 religions and 3 education levels) allows me to highlight their positive or negative interactions for happiness and health; obviously, religious ethics represent alternative *contexts*, whereas secular ethics depict alternative *policies*.

Finally, I use data on observed phenomena at the country level (i.e., religion percentages and majorities, gross enrolments, per capita and per-student education expenditures, per capita health expenditures, GDP, Gini index) rather than at the individual level; thus, some individual characteristics will be disregarded (e.g., gender, age, work status, marital status, specific beliefs or precepts, degree of religiosity, religious service attendance), while some social characteristics will be emphasised (e.g., the shared religious individual precepts and social networks; the shared secular individual knowledge and social norms) by introducing an *additional* assumption (e.g., some intercultural interactions are negligible, since they are unrealistic at the world level if one country identifies with one culture) to obtain additional insights.

Note that LS is the only variable calculated by averaging information obtained by surveys at the individual level (i.e., each individual can properly assess his or her life satisfaction only) by adopting a representative individual approach at the country level (i.e., the multilevel approach is problematic due to the regional differences within each country) (e.g., [23, 25]). Indeed, LS is the proper variable with which to estimate the impacts of ethics (i.e., it measures whether life is worthwhile rather than happy).

The *contribution* of this paper is the movement forwards from estimating the marginal impacts of EDU or REL on happiness or health as unsatisfactory (i.e., religious and secular ethics have crucial consequences on individuals’ lives) by referring to a dynamic theoretical model to examine whether the parameter values estimated by statistics are consistent with a convergence towards nonlinear and long-run equilibria of happiness and health (i.e., existence and stability of a steady-state equilibrium are essential to attach a meaning to the marginal impacts of EDU or REL on happiness or health). All results (i.e., in any context and for any policy) support the expected existence and stability of long-run equilibria of happiness and health (e.g., the 4-year lag for the short-run indirect impact of happiness on health at approximately 3 HLEB for each 1 out of 10 LS; the oscillating dynamics towards the long-run equilibrium in 8 years). Actually, the prevailing long-run equilibria of happiness and health depend on contexts and policies, although there is no religious or secular ethics with beneficial impacts on both happiness and health at both the individual and social levels.

### Model

Within an *each* individual perspective, Zagonari [27] represents the dynamic interrelationship between happiness (*hap*[*t*]) and health (*hea*[*t*]) at each time *t* by using two dynamic equations for an individual’s achievements (*y*[*t*]), in which standardisations are applied to the original family income *fy* and to the individual’s original health *fh*, while parameters are represented by the reference group’s average achievement *ay*, the education level *ed*, the feasible set for opportunities *os*, the ethical freedom *fr*, the number of past periods that affect the current health *me*, the occupation type *oc*, and the employment status *em*:

hap[t]=α{(y[t]–fs)/fs}+β{(y[t]–y[t–1])/y[t–1]}+γ{(y[t]–ay)/ay}+hea[t] (1)

$$hea[t]=os+{\sum_{t–me}}^{t–1}hap[t]+y[t]+em+ed+oc$$ (2)

where:

fs=fy+fh–u[t]+fr;oc≤0,em≥0,me≥1;andu[t]isin[–u*,+u*]

where α represents Aristotle’s contribution to happiness (achievements with respect to the individual’s opportunity set *fs*), β represents Epicurus’ contribution (short-run achievements), γ represents Zeno’s contribution (achievements with respect to the individual’s reference group), such that α + β + γ = 1, *u*[*t*] is the personal uncertainty, and u* is the long-run equilibrium uncertainty. Note that I will refer to Eqs 1 and 2 as “the life model” by using capital letters to stress that I am moving from a theoretical to an empirical model.

The *representative* individual perspective at the country level justified in the Literature section requires some adjustments. In particular, some variables will be neglected, although they are theoretically relevant to estimating the long-run equilibrium for each single individual (e.g., the original family income *fy*, the individual’s original health *fh*, the feasible set for opportunities *os*, the ethical freedom *fr*); some features will be represented by alternative variables (e.g., Aristotle’s contribution to happiness will be captured by referring to gross enrolment and per-student expenditure in secondary education, Epicurus’ contribution to happiness will be captured by introducing the one year lag of GDP, and Zeno’s contribution to happiness will be captured by comparing the mean and median of LS); some variables will be disregarded as irrelevant to estimating the long-run equilibrium for the average individual at the country level (e.g., the personal uncertainty); and some features could be introduced in future research (e.g., occupation type and employment status by introducing employment rates in at least three production sectors).

Thus, I will refer to the two-equation statistical dynamic model as follows:

$${LS}_{i,t}=\eta_{0}+\eta_{1}LnGDP_{i,t}+\eta_{2}{GINI}_{i,t}+\eta_{3}HL{EB}_{i,t}+\eta_{4}REL_{i,t}-\eta_{5}EDU_{i,t}+\eta_{6}ADD_{i,t}+\varepsilon_{i,t}$$ (3)

$${HLEB}_{i,t}=\theta_{0}+{\theta_{1}LnGDP}_{i,t}-\theta_{2}{GINI}_{i,t}+\theta_{3}\sum_{s=t-1-k}^{t-1}(\frac{1}{k})LS_{i,s}-\theta_{4}{REL}_{i,t}+\theta_{5}{EDU}_{i,t}+{\theta_{6}ADD}_{i,t}+\zeta_{i,t}$$ (4)

where positive or negative signs are based on the literature results. Furthermore, CON = constant; LnGDP = the natural logarithm of GDP per capita [$ PPP]; GINI = the Gini index [0, 65]; LS = life satisfaction [0, 10]; HLEB = healthy life expectancy at birth (years); REL = BUDM, CHRM, HINM, ISLM, JUDM as dummy variables with a value of 1 if a religion is a 50% majority and 0 if it is not a majority in social regressions, and REL = BUDP, CHRP, HINP, ISLP, JUDP in percentages in individual regressions; EDU = gross enrolment in primary, secondary and tertiary education (i.e., GEP, GES, GET) in percentages in social regressions, and EDU = *government* EDU expenditures in primary, secondary and tertiary education (i.e., EEP, EES, EET) per student [$ PPP] in individual regressions; and ADD = *government* total EDU expenditures per capita [$ PPP] and *total* health expenditures per capita [$ PPP].

Note that I perform regressions with HLEB replaced by LEB to compare my results with the health literature at the social level (see S9-S12 Tables in S1 File). Moreover, I perform regressions with alternative values of k (i.e., 3, 4 and 5) to evaluate the most significant time lag for the impacts of LS on HLEB. Finally, I perform regressions with the lagged GDP together with the contemporaneous GDP to compare my results with the happiness literature at the individual level (see S21-S24 Tables in S1 File).

In particular, I summarise positive and negative significant *marginal* coefficients for both EDU policies and REL ethics to look for a possible dominant secular or religious ethics at the social or individual level in terms of happiness and health. Moreover, I compare *average* significant *marginal* coefficients in terms of sign and size to highlight potential interactions between REL and EDU. Finally, I sum *average* significant coefficients from EDU (i.e., impacts of primary, secondary and tertiary education) for the intercultural representative individual at the world level in both religious contexts (i.e., countries with and without a majority religion) to look for a possible dominant choice of EDU levels in terms of happiness and health from an *each* individual perspective.

### Data

The two-equation statistical dynamic model specified in in the Model section suggests using the annual dataset from World Happiness Reports, integrated by World Health Organisation data on HLEB and by World Value Survey data on LS. In particular, LS is consistently measured across nations (e.g., [35, 36]), since it is based on surveys using a single question (i.e., “all things, considered, how are you satisfied or dissatisfied with your life as-a-whole these days on a scale from 0 to 10?”), where cultural differences in the type of information individuals use when making life-satisfaction judgments (i.e., the personally important domains of life and the main interpretation of overall life) are caught by their answers (e.g., [37, 38]). Similarly, HLEB consistently measures fully health (e.g., [39] Velasco, 2022), where mental health included in HLEB does not allow me to distinguish the effects of EDU and REL on physical and mental health (e.g., [40]). Moreover, I refer to the World Bank and World Religions datasets for other variables. Finally, linear interpolations between data in different years for the same country are applied if some data are missing. In particular, this interpolation method is similar to the item-level imputation for a linear growth model suggested by Enders [41]; it is adequate in my context because it does not imply linear dependence between parameters across panels, since I replace some missing data for each panel separately; the resulting dataset is used to estimate linear relationships; and it provides unbiased parameter estimates and standard errors. In other words, instead of making assumptions about the data distribution to obtain the missing data, I replace missing data under the assumption that they represent a linear growth model. The final dataset consists of 162 countries across 21 years (2000–2020), with a total number of 3402 observations.

Table 2 provides the main statistics of the variables used. Note that the median of LS is smaller than its mean. Moreover, the number of countries with a majority religion are 8, 90, 2, 46 and 1 for BUD, CHR, HIN, ISL, JUD, respectively. In particular, these 5 religions represent 87% of the world’s population. Finally, the median of HLEB is larger than its mean. It is important to stress that the present paper uses happiness (i.e., an informal concept) or LS (i.e., a proper statistical measure of happiness for a life model) and health (i.e., an informal concept) or HLEB (i.e., a proper statistical measure of health for a life model) interchangeably to improve its readability, although these terms refer to different dimensions of subjective well-being and health status, respectively.

**Table 2:** Summary statistics.

		Mean	SD	Max	Min	Median
LS	A ladder in [0, 10]	5.41	2.78	8.02	2.38	**5.20**
HLEB	Years	62	31	77	32	64
LEB	Years	70	35	85	39	**72**
GDP	Per capita $ PPP	16,990	17,334	14,1635	435	9,850
GINI	An index in [0, 0.99]	38.52	19.72	64.80	23.20	37.81
BUD	Percentage	0.05	0.13	0.87	0.00	
CHR	Percentage	0.50	0.39	0.99	0.00	
HIN	Percentage	0.02	0.07	0.74	0.00	
ISL	Percentage	0.29	0.33	1.00	0.00	
JUD	Percentage	0.01	0.05	0.74	0.00	
GEP	Percentage	102.34	51.19	150.79	20.88	102.47
GES	Percentage	77.53	44.62	163.93	5.93	84.56
GET	Percentage	35.60	27.98	148.53	0.20	29.92
EEP	Per student Thousand $ PPP	2.952	3.175	23.203	13	1.322
EES	Per student Thousand $ PPP	3.542	3.691	22.872	34	1.675
EET	Per student Thousand $ PPP	7.442	9.853	105.095	1	3.860
EE	Per capita $ PPP	791	840	5995	7	407
HE	Per capita $ PPP	1170	1297	10921	7	591

## Results

The use of the time-dependent variables identified in the Data section (i.e., LS and HLEB) suggests checking for unit roots (e.g., Islam, 2020). S1 and S2 Tables in S1 File confirm that both LS and HLEB converge in 8 years. Thus, to keep a satisfactory number of observations, I fix k at 4 and calculate the average of the previous k values for LS (LSk4) to be used in Eq (4). S13-S20 Tables in S1 File support this approach, since R^2^ shows the largest values where REL takes a social perspective (i.e., 0.60 and 0.73 in Table 3), and R^2^ shows the same values where REL takes an individual perspective (i.e., 0.60 and 0.72 in Table 4, 0.61 and 0.72 in Tables 5 and 6).

**Table 3:** RELs & EDUs. R^2^ (LS) = 0.60, R^2^ (HLEB) = 0.73. Consistent and robust estimates are obtained by applying a three-stage least square (3SLS) estimator to a balanced sample (i.e., 162 countries across 21 years); LSk4 is the average of the previous 4 year values for LS.

		Coef.	Std. Err.	z	P>z	[95% Conf. Interval]	
LS							
	lnGDP	.3064404	.0195803	15.65	0.000	.2680637	.3448171
	GINI	.0043219	.0018201	2.37	0.018	.0007545	.0078893
	HLEB	.0857813	.002916	29.42	0.000	.080066	.0914966
	BUDM	-.0827249	.0657642	-1.26	0.208	-.2116203	.0461705
	CHRM	.4139106	.0426604	9.70	0.000	.3302979	.4975234
	HINM	.2180225	.1138638	1.91	0.056	-.0051466	.4411915
	ISLM	.0561106	.0455312	1.23	0.218	-.033129	.1453502
	JUDM	.923416	.1546504	5.97	0.000	.6203068	1.226525
	GEP	-.0105601	.0008982	-11.76	0.000	-.0123206	-.0087996
	GES	.0006183	.000851	0.73	0.467	-.0010496	.0022862
	GET	-.0057967	.000735	-7.89	0.000	-.0072372	-.0043562
	CONS	-1.841833	.1864447	-9.88	0.000	-2.207258	-1.476408
HLEB							
	lnGDP	1.228951	.1218617	10.08	0.000	.9901068	1.467796
	GINI	-.0958014	.0107498	-8.91	0.000	-.1168707	-.0747322
	LSk4	3.113357	.1105013	28.17	0.000	2.896779	3.329936
	BUDM	1.370708	.3929138	3.49	0.000	.6006113	2.140805
	CHRM	-1.609453	.2585379	-6.23	0.000	-2.116178	-1.102728
	HINM	-.1081894	.6824546	-0.16	0.874	-1.445776	1.229397
	ISLM	-.5516751	.2725288	-2.02	0.043	-1.085822	-.0175284
	JUDM	-.5346414	.9333037	-0.57	0.567	-2.363883	1.2946
	GEP	.0583418	.0054009	10.80	0.000	.0477563	.0689273
	GES	.0507417	.0049956	10.16	0.000	.0409505	.0605328
	GET	.0537461	.0043178	12.45	0.000	.0452834	.0622087
	CONS	26.47924	1.005573	26.33	0.000	24.50835	28.45013

**Table 4:** RELi & EDUi. R^2^ (LS) = 0.60, R^2^ (HLEB) = 0.72. Consistent and robust estimates are obtained by applying a three-stage least square (3SLS) estimator to a balanced sample (i.e., 162 countries across 21 years); LSk4 is the average of the previous 4 year values for LS.

		Coef.	Std. Err.	z	P>z	[95% Conf. Interval]	
LS							
	lnGDP	.0403637	.0236785	1.70	0.088	-.0060452	.0867726
	GINI	.0114058	.0018951	6.02	0.000	.0076914	.0151202
	HLEB	.0861713	.0028747	29.98	0.000	.080537	.0918056
	BUDP	-.0994286	.1195767	-0.83	0.406	-.3337946	.1349373
	CHRP	.3950921	.0842128	4.69	0.000	.2300381	.5601461
	HINP	.6129055	.1476382	4.15	0.000	.3235399	.9022712
	ISLP	.170761	.079773	2.14	0.032	.0144088	.3271131
	JUDP	1.162371	.2194625	5.30	0.000	.7322327	1.59251
	EEP	.004725	.0116454	0.41	0.685	-.0180996	.0275497
	EES	.0603684	.0107534	5.61	0.000	.0392921	.0814448
	EET	.0036683	.001471	2.49	0.013	.0007852	.0065515
	CONS	-1.226336	.1770549	-6.93	0.000	-1.573357	-.8793146
HLEB							
	lnGDP	4.041061	.1187563	34.03	0.000	3.808303	4.273819
	GINI	-.1429581	.0112496	-12.71	0.000	-.1650068	-.1209093
	LSk4	3.233527	.1117647	28.93	0.000	3.014472	3.452582
	BUDP	2.4919	.7240369	3.44	0.001	1.072814	3.910987
	CHRP	-.831695	.5134358	-1.62	0.105	-1.838011	.1746207
	HINP	-2.228604	.898774	-2.48	0.013	-3.990169	-.4670398
	ISLP	-.9146806	.4844517	-1.89	0.059	-1.864188	.0348272
	JUDP	-.6081595	1.340684	-0.45	0.650	-3.235851	2.019532
	EEP	.3682409	.0702906	5.24	0.000	.2304738	.506008
	EES	-.4924509	.0649681	-7.58	0.000	-.6197861	-.3651157
	EET	-.1230754	.0085803	-14.34	0.000	-.1398925	-.1062582
	CONS	15.09239	1.042495	14.48	0.000	13.04913	17.13564

**Table 5:** RELi & EDUs. R^2^ (LS) = 0.61, R^2^ (HLEB) = 0.72. Consistent and robust estimates are obtained by applying a three-stage least square (3SLS) estimator to a balanced sample (i.e., 162 countries across 21 years); LSk4 is the average of the previous 4 year values for LS.

		Coef.	Std. Err.	z	P>z	[95% Conf. Interval]	
LS							
	lnGDP	.3049576	.0196773	15.50	0.000	.2663908	.3435244
	GINI	.0050731	.001847	2.75	0.006	.001453	.0086932
	HLEB	.0857408	.0029633	28.93	0.000	.0799328	.0915488
	BUDP	-.3962829	.1198036	-3.31	0.001	-.6310935	-.1614722
	CHRP	.2834228	.0840418	3.37	0.001	.1187039	.4481417
	HINP	.3395104	.150144	2.26	0.024	.0452336	.6337871
	ISLP	-.1599931	.0792683	-2.02	0.044	-.315356	-.0046302
	JUDP	1.003312	.2175787	4.61	0.000	.5768658	1.429759
	GEP	-.0109379	.0008999	-12.15	0.000	-.0127017	-.009174
	GES	.000837	.0008558	0.98	0.328	-.0008403	.0025143
	GET	-.0058005	.000757	-7.66	0.000	-.0072841	-.0043169
	CONS	-1.673609	.1956538	-8.55	0.000	-2.057084	-1.290135
HLEB							
	lnGDP	1.285733	.1203556	10.68	0.000	1.049841	1.521626
	GINI	-.101651	.0107568	-9.45	0.000	-.122734	-.080568
	LSk4	3.013878	.1088944	27.68	0.000	2.800449	3.227307
	BUDP	4.924426	.7025816	7.01	0.000	3.547391	6.30146
	CHRP	-.1537398	.4980493	-0.31	0.758	-1.129898	.8224189
	HINP	-.6936127	.8886547	-0.78	0.435	-2.435344	1.048119
	ISLP	.9904331	.468389	2.11	0.034	.0724075	1.908459
	JUDP	1.538791	1.292057	1.19	0.234	-.9935942	4.071177
	GEP	.0590945	.0053441	11.06	0.000	.0486202	.0695687
	GES	.0499777	.0049627	10.07	0.000	.0402511	.0597044
	GET	.0539374	.0043893	12.29	0.000	.0453345	.0625404
	CONS	25.28592	1.058082	23.90	0.000	23.21212	27.35972

**Table 6:** RELs & EDUi. R^2^ (LS) = 0.61, R^2^ (HLEB) = 0.72. Consistent and robust estimates are obtained by applying a three-stage least square (3SLS) estimator to a balanced sample (i.e., 162 countries across 21 years); LSk4 is the average of the previous 4 year values for LS.

		Coef.	Std. Err.	z	P>z	[95% Conf. Interval]	
LS							
	lnGDP	.0486883	.0235493	2.07	0.039	.0025324	.0948441
	GINI	.0102602	.0018748	5.47	0.000	.0065856	.0139348
	HLEB	.0855758	.0028453	30.08	0.000	.0799991	.0911525
	BUDM	.0109795	.0656384	0.17	0.867	-.1176695	.1396284
	CHRM	.3650706	.0424569	8.60	0.000	.2818566	.4482847
	HINM	.104641	.1127042	0.93	0.353	-.1162552	.3255372
	ISLM	.1954014	.0459599	4.25	0.000	.1053217	.2854812
	JUDM	.9774281	.1563104	6.25	0.000	.6710653	1.283791
	EEP	-.0039941	.011603	-0.34	0.731	-.0267356	.0187474
	EES	.0631351	.0107401	5.88	0.000	.0420848	.0841854
	EET	.0036957	.0014612	2.53	0.011	.0008319	.0065595
	CONS	-1.207648	.1679655	-7.19	0.000	-1.536854	-.8784414
HLEB							
	lnGDP	4.037765	.1196367	33.75	0.000	3.803281	4.272248
	GINI	-.1360881	.0112475	-12.10	0.000	-.1581328	-.1140434
	LSk4	3.283188	.11314	29.02	0.000	3.061438	3.504938
	BUDM	.5345573	.4019733	1.33	0.184	-.2532959	1.322411
	CHRM	-1.076862	.2635809	-4.09	0.000	-1.593472	-.5602532
	HINM	.8294179	.6905268	1.20	0.230	-.5239898	2.182826
	ISLM	-1.075826	.2820378	-3.81	0.000	-1.62861	-.5230424
	JUDM	-1.766156	.9655384	-1.83	0.067	-3.658577	.1262642
	EEP	.4170994	.0705822	5.91	0.000	.2787608	.5554381
	EES	-.521436	.0654612	-7.97	0.000	-.6497377	-.3931343
	EET	-.1257145	.0085895	-14.64	0.000	-.1425497	-.1088793
	CONS	14.81959	.9967973	14.87	0.000	12.86591	16.77328

Moreover, the use of the current values of GDP and the Gini Index, together with the per capita health expenditure and the per capita education expenditure, in both Eqs (3) and (4) to clean the impacts of religious and secular ethics from differences in income and inequality, together with differences in the effectiveness of health care and education systems, suggests using levels rather than changes in GDP (e.g., [6]). Indeed, this approach is consistent with the *representative* individual perspective at the country level.

Finally, Eqs 3 and 4 (i.e., the dynamic relationships between LS and HLEB and between HLEB and LSk specified in the Model section) identify a structural form of a simultaneous equation system based on the theory of reciprocal causality discussed in the Model section (i.e., Eqs 1 and 2); for significant estimated parameters, the literature (e.g., [42, 43]) speaks of association or causality as well as of linkages or impacts. Next, the Dumitrescu and Hurlin test for Granger noncausality in S3 and S4 Tables in S1 File reject “no causality” in favour of “causality for at least one panel” for both HLEB on LS and LS on HLEB (i.e., both *P* values smaller than 0.001). It is important to stress that the present paper uses the terms linkage and link as synonyms of the term association to avoid its ambiguity, since the revealed statistical relationships are associational.

Note that the use of HLEB at time t-1 as an instrumental variable for HLEB at time t in Eq (3) does not affect its positive sign and its significance level (see S5-S8 Tables in S1 File), and the use of LSk5 as an instrumental variable for LSk4 in Eq (4) does not affect its positive sign and its significance level (see S17- S20 Tables in S1 File). Similarly, reverse causality from happiness and health to EDU is meaningless (i.e., LS and HLEB refer to people who choose EDU many years before), and reverse causality from happiness and health to REL is meaningless (i.e., the choice of REL depends on social contexts rather than on individual happiness level or health status).

Table 3 presents the 3SLS regressions for both EDU and REL with a social perspective, Table 4 presents the 3SLS regressions for both EDU and REL with an individual perspective, Table 5 presents the 3SLS regressions where REL takes an individual perspective and EDU takes a social perspective, and Table 6 presents the 3SLS regressions where REL takes a social perspective and EDU takes an individual perspective.

Note that a 3SLS estimator applied to sufficiently informative samples, in general, and to a sufficiently large sample (i.e., 162 countries across 21 years), in particular, produces consistent and robust estimates of structural parameters by correcting for simultaneity between the endogenous variables and the disturbance terms of the statistical model.

In particular, I do not use a vector error correction model for two reasons. First, my focus is on the short-run impacts of the current HLEB on LS and the lagged LS on current HLEB (i.e., η_3_ and θ_3_) for *all* panels in alternative contexts rather than the long-run relationship between LS and HLEB; a common balance at the world level between the happiness dimension in Eq 3 and the health dimension in Eq 4 would be hardly interpreted within the fields of moral philosophy and theology, and it is actually rejected by the estimated parameters. Second, LS and HLEB are stationary for *all* panels (i.e., the Levin-Lin-Chu test for LS and HLEB in S1 and S2 Tables in S1 File rejects the hypothesis that the “panels contain unit roots”), but *some* panels are not cointegrated; the Kao test does not reject “no cointegration” in favour of “*all* panels are cointegrated” (i.e., *P* value at 0.076), and the Westerlund test rejects “no cointegration” in favour of “*some* panels are cointegrated” (i.e., *P* value smaller than 0.001).

Note that coefficients for REL (both majority and minority religions) represent impacts with respect to atheism or other religions. Moreover, error terms are *not* assumed to be independent across Eqs (3) and (4), with the related consequences on the significance and size of each variable coefficient. Indeed, I do not use the “independent correlation structure” option available in the Stata software package to force it to treat the covariance matrix of equation disturbances as diagonal when estimating model parameters (i.e., I obtain more efficient estimations with 3SLS). Finally, coefficients for EDU (both gross enrolment and per-student expenditure) represent impacts with respect to illiteracy.

### Linkages

The main *specific* insights can be summarised as follows.

Table 3 suggests that REL is good for happiness with regard to CHR, HIN and JUD, while REL is good for health with regard to BUD, but it is bad with regard to CHR and ISL (e.g., a belief in miracles which reduces prognostic understanding in [44]; a focus on afterlife which leads to a lower sense of personal control and a smaller attention to physical health behaviours in [45]; a willingness to defer to God’s will which reduces the life extension desirability in [46]). EDU is bad for happiness with regard to primary and tertiary education (e.g., a clearer perception of negative consequences from future problematic scenarios in [47]; a smaller amount of free time in [48]; a smaller job satisfaction due to a change in individuals’ subjective evaluation of their conditions and expectations in [49]), while EDU is good for health.

Table 4 suggests that REL is good for happiness with regard to CHR, HIN, ISL and JUD, while REL is bad for health with regard to HIN and ISL (it is good with regard to BUD). EDU is good for happiness with regard to secondary and tertiary education, while EDU is good for health with regard to primary education (it is bad for secondary and tertiary education).

Table 5 suggests that REL is good for happiness with regard to CHR, HIN and JUD (it is bad with regard to BUD and ISL), while REL is *not* bad for health (it is good with regard to BUD and ISL). EDU is bad for happiness with regard to primary and tertiary education, while EDU is good for health.

Table 6 suggests that REL is good for happiness with regard to CHR, ISL and JUD, while REL is bad for health with regard to CHR, JUD and ISL. EDU is good for happiness with regard to secondary and tertiary education, while EDU is good for health with regard to primary education (it is bad with regard to secondary and tertiary education).

The main *general* insights can be summarised as follows.

1. The significant and positive association of LnGDP with both LS and HLEB in all regressions is consistent with the literature based on representative individuals at the country level. Note that S21-S24 Tables in S1 File support the use of estimations within an each individual perspective at the country level since an increase in income has a positive link with both happiness and health, apart from countries where social secular ethics are well established (i.e., Epicurus’s contribution to happiness).
2. The significant and negative association of the GINI index with HLEB in all regressions seems to represent inequalities in access to health care, whereas the significant and positive association of the GINI index with LS in all regressions seems to suggest that people obtain satisfaction from being better off than their reference groups (i.e., Zeno’s contribution to happiness). Indeed, the reference to *each* individual in the theoretical model is translated into the *average* individual for each country in the statistical model, and the mean of LS at 5.41 is larger than the median of LS at 5.20. Note that the significant and nonnegative linkages of both gross enrolment and per-student expenditure only for secondary education support the use of estimations within an each individual perspective (i.e., Arisotle’s contribution to happiness).
3. A year increase in HLEB increases LS by approximately 0.086 in all contexts, while a unitary increase in LS increases HLEB by approximately 3.161 years in all contexts. Note that the latter link for the intercultural representative individual at the world level (i.e., 3.161 x 5.41/62 = 0.27) is similar to the reduction of risk of death over the follow-up period for happy people (i.e., - 20%) estimated in US [50]
4. Comparing REL in majorities and percentages in Table 9 (i.e., links in Table 3 *minus* links in Table 5 at given GE; links in Table 6 *minus* links in Table 4 at given EE) suggests that religious social ethics have a more *beneficial* effect on happiness than that of religious individual ethics if coupled with social capital (i.e., 0 > mean of REL links in I column = -0.014 > 0 > mean of REL links in III column = -0.038), whereas religious social ethics have a less *detrimental* effect on health than that of religious individual ethics if coupled with individual knowledge (i.e., 0 > mean of REL links in III column = 0.134 > mean of REL links in I column = -1.341)
5. Comparing EDU in terms of gross enrolment and per-student expenditures in Table 9 (i.e., links in Table 3 *minus* links in Table 6 at given MAJ REL; links in Table 5 *minus* links in Table 4 at given MIN REL) suggests that secular individual ethics have a similar *detrimental* effect on happiness as that of secular social ethics (i.e., mean of EDU links in II column = -0.023 ≈ mean of EDU links in IV column = -0.028), whereas secular social ethics have a similar *beneficial* effect on health as that of secular individual ethics (i.e., mean of EDU links in II column = 0.137 ≈ mean of EDU links in IV column = 0.131)

Note that REL could have a positive indirect impact on health with a temporal lag by increasing happiness.

Table 7 compares signs of significant linkages of EDU with happiness and health from social and individual perspectives. This suggests that there is no EDU *policy* with a positive link with both happiness and health for all EDU levels. In particular, a positive link with happiness is coupled with a negative link with health (e.g., EES, EET), whereas a positive link with health is coupled with a negative link with happiness (e.g., GEP, GET). In other words, as for the social and individual components of health and happiness, *social capital* from EDU prevails for health (i.e., increasing gross enrolment rates in primary, secondary and tertiary education implies an increased *average* health at a country level, whereas increasing per-student per-year education expenditures in secondary and tertiary education does not), whereas *individual knowledge* from EDU prevails for happiness (i.e., increasing per-student per-year education expenditures in secondary and tertiary education implies an increased *average* happiness at a country level, whereas increasing gross enrolment rates in primary and tertiary education does not).

**Table 7:** Linkages of social and individual EDU policies. GE = gross enrolment refers to social capital (SOC) in both Tables 3 and 5; EE = per-student education expenditure refers to individual knowledge (IND) in both Tables 4 and 6;— = negative significant link; + = positive significant link; 0 = no significant link.

	Happiness		Health	
	SOC (GE)	IND (EE)	SOC (GE)	IND (EE)
Primary	-	0	+	+
Secondary	0	+	+	-
Tertiary	-	+	+	-

Table 8 compares signs of significant linkages of REL on happiness and health from social and individual perspectives. This suggests that there is no REL *ethics* with a positive link with both happiness and health at both social and individual levels. In particular, a positive link with happiness is coupled with a negative link with health, with BUD and HIN characterised by a larger individual perspective and CHR, JUD and ISL characterised by a larger social perspective. In other words, as for the social and individual components of health and happiness, *social capital* from EDU prevails for happiness in countries where CHR, HIN and JUD are minority religions, whereas *social capital* from EDU prevails for health in countries where BUD and ISL are minority religions.

**Table 8:** Linkages of social and individual REL ethics. GE = gross enrolment refers to social capital (SOC) in both Table 3 with a majority religion (MAJ) and Table 5 with a minority religion (MIN); EE = per-student education expenditure refers to individual knowledge (IND) in both Table 4 with a minority religion (MIN) and Table 6 with a majority religion (MAJ);— = negative significant link; + = positive significant link; 0 = no significant link.

	Happiness				Health			
	SOC (MAJ)		IND (MIN)		SOC (MAJ)		IND (MIN)	
	GE	EE	GE	EE	GE	EE	GE	EE
BUD	0	0	-	0	+	0	+	+
CHR	+	+	+	+	-	-	0	0
HIN	+	0	+	+	0	0	0	-
ISL	0	+	-	+	-	-	+	-
JUD	+	+	+	+	0	-	0	0

Table 9 compares the sizes of the significant linkages of REL if coupled with social or individual EDU policies and compares the sizes of the significant linkages of EDU if coupled with social or individual REL ethics. This suggests that the linkages of EDU policies do not depend on REL contexts, while the linkages of REL social and individual ethics might depend on EDU policies. Let me limit my remarks to links with LS larger than 0.2 (out of its mean at 5.41) and to links with HLEB larger than 2 (out of its mean at 62). In particular, there are no differences in the linkages of gross enrolment and per-student expenditure in majority vs. minority religious contexts (i.e., in Column I, which is based on Table 3 *minus* Table 5, the means of EDU links is 0.000 for happiness and 0.000 for health; in Column III, which is based on Table 6 *minus* Table 4, the means of EDU links is 0.001 for happiness and 0.006 for health). In contrast, in minority religion contexts (i.e., in Column II, which is based on Table 5 *minus* Table 4, the means of REL links is -0.175 for happiness and 0.868 for health; in Column IV, which is based on Table 3 *minus* Table 6, the means of REL links is -0.040 for happiness and 0.272 for health), if individual knowledge is provided by the governmental education system, then Buddhist, Hindus and Muslim individuals are happier (i.e., there is a positive interaction between *individual* secular and religious ethics, since -0.175 < -0.040 < 0), while if social capital is provided by the governmental education system, then Buddhist and Jewish individuals are healthier (i.e., there is a positive interaction between *social* secular and religious ethics, since 0.868 > 0.272 > 0). In summary, secular individual knowledge is complementary to minority religions for happiness, while secular social capital is complementary to minority religions for health [51], with smaller complementary linkages for majority religions.

**Table 9:** Interactions between social and individual REL ethics and social and individual EDU policies as differences between significant social linkages *minus* individual linkages. Grey cells should *not* be analysed autonomously (i.e., they should be compared with other grey cells), since they provide differences of coefficients expressed in different units (i.e., 0 or 1 values with percentages in [0,1] and percentages of gross enrolments with per-student expenditures in $).

	Table 3 –Table 5	Table 5 –Table 4	Table 6 –Table 4	Table 3 –Table 6
	ΔREL if GE	ΔEDU if MIN REL	ΔREL if EE	ΔEDU if MAJ REL
LS				
lnGDP	0.001	0.265	0.008	0.258
GINI	-0.001	-0.006	-0.001	-0.006
HLEB	0.000	0.000	-0.001	0.000
BUD	0.000	0.000	0.000	0.000
CHR	0.130	-0.112	-0.030	0.049
HIN	-0.121	-0.273	0.000	0.000
ISL	0.000	-0.331	0.025	-0.195
JUD	-0.080	-0.159	-0.185	-0.054
EDUP	0.000	0.000	0.000	-0.011
EDUS	0.000	-0.060	0.003	-0.063
EDUT	0.000	-0.009	0.000	-0.009
CONS	-0.168	-0.447	0.019	-0.634
HLEB				
lnGDP	-0.057	-2.755	-0.003	-2.809
GINI	0.006	0.041	0.007	0.040
LSk4	0.099	-0.220	0.050	-0.170
BUD	-3.554	2.433	0.000	1.371
CHR	-1.456	0.000	0.832	-0.533
HIN	0.000	0.000	0.000	0.000
ISL	-1.542	1.905	-0.161	0.524
JUD	0.000	0.000	0.000	0.000
EDUP	-0.001	-0.309	0.049	-0.359
EDUS	0.001	0.542	-0.029	0.572
EDUT	0.000	0.177	-0.003	0.179
CONS	1.193	10.194	-0.273	11.660

If linkages between all education levels and both HLEB and LS are assumed to be independent (e.g., the positive linkages of individual knowledge from tertiary education with health for the intercultural representative individual at global level do not include the positive linkages of individual knowledge from primary education with health) and if interactions between people with different education levels are assumed to be negligible (e.g., the negative linkages of individual knowledge from tertiary education with happiness for the intercultural representative individual at global level do not arise from the exploitation of people with a primary and secondary education by people with a tertiary education), it is possible to adopt an *each* individual perspective (i.e., the statistical associations highlighted *on average* for the intercultural representative individual are used to obtain insights *on average* from the analytical model within an each individual perspective). In particular, the sum of coefficient values of primary, secondary and tertiary EDU for LS in religious majority contexts are -1.09, -0.82 and -1.00, respectively, whereas in religious minority contexts, they are -1.11, -0.83 and -1.01, respectively. The sum of coefficient values of primary, secondary and tertiary EDU for HLEB in religious majority contexts are 7.20, 9.29 and 10.27, respectively, whereas in religious minority contexts, they are 7.14, 9.27 and 10.27, respectively.

**Fig 1:**
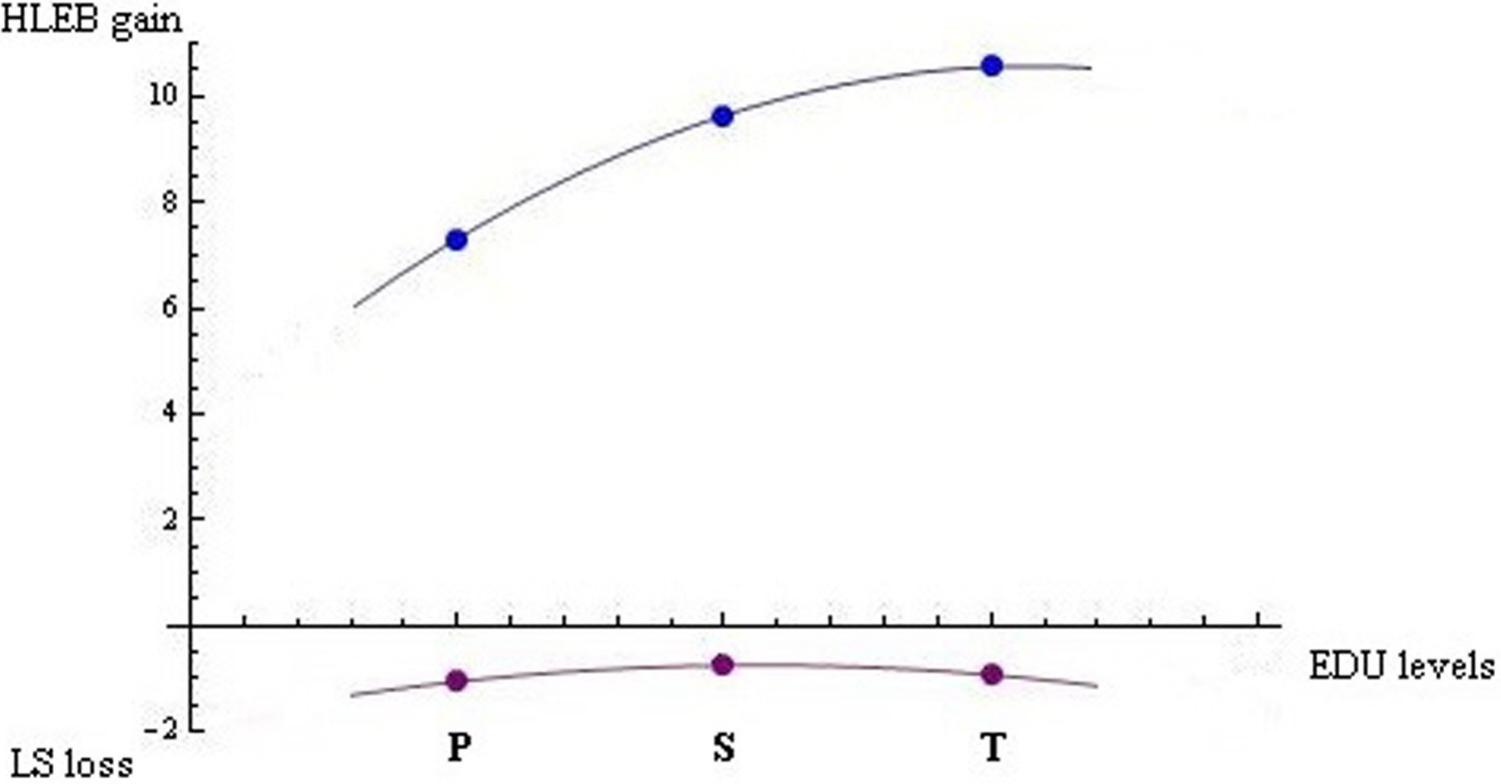
HLEB gains in years and LS losses in [0, 10] from primary, secondary and tertiary EDU (P, S, and T, respectively) within an each individual perspective. Points are based on estimations, whereas curves are based on quadratic interpolations of points.

Fig 1 shows gains in health (years) and losses in happiness (0 to 10 scores) from education choices within an *each* individual perspective based on *average* coefficients (i.e., Fig 1 does not depict the actual situation of each single person with regard to HLEB and LS). Thus, EDU levels increase health at a decreasing rate, whereas they decrease happiness to a greater extent at the primary and tertiary levels.

**Fig 2:**
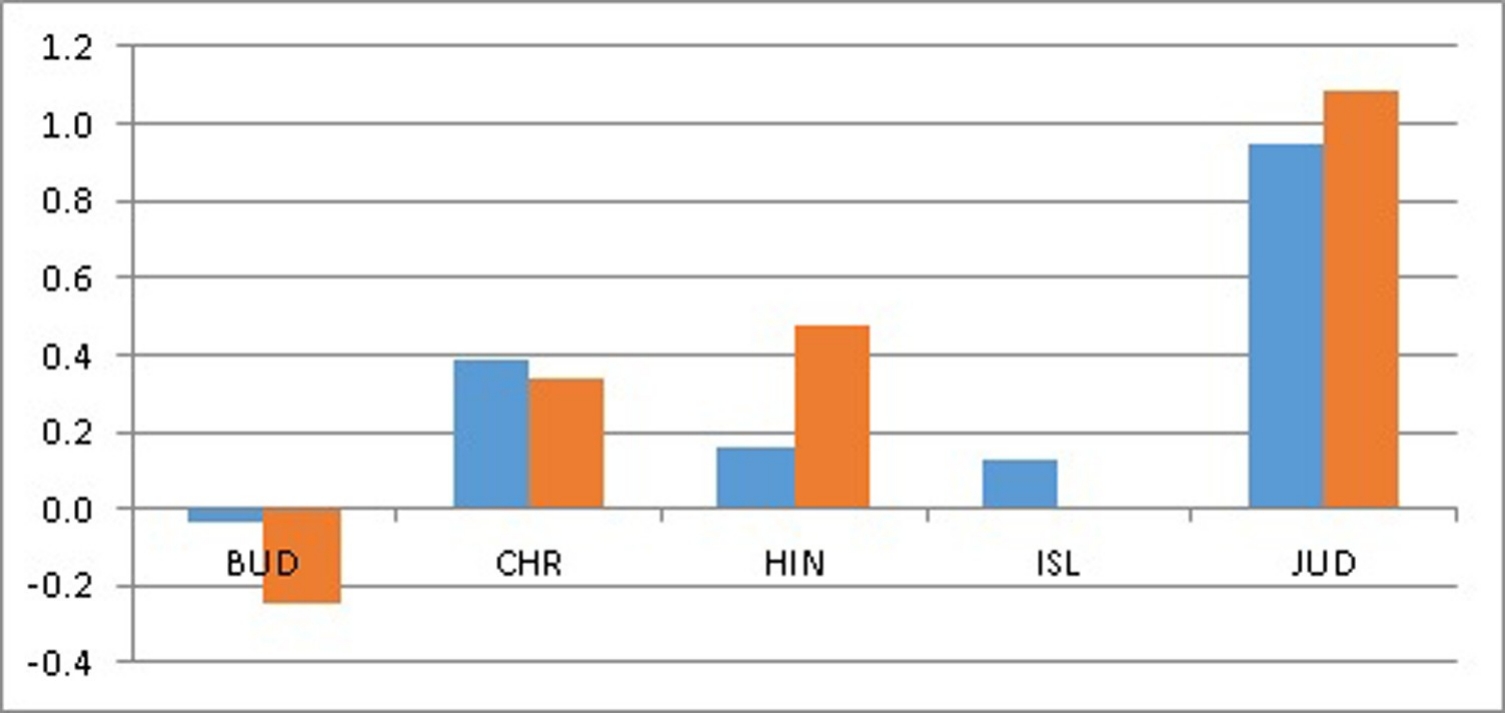
LS gains (positive values) and losses (negative values) in [0, 10] from Buddhism, Christianity, Hinduism, Islam, and Judaism as majority religions (blue bars) and minority religions (red bars) within an each individual perspective.

**Fig 3:**
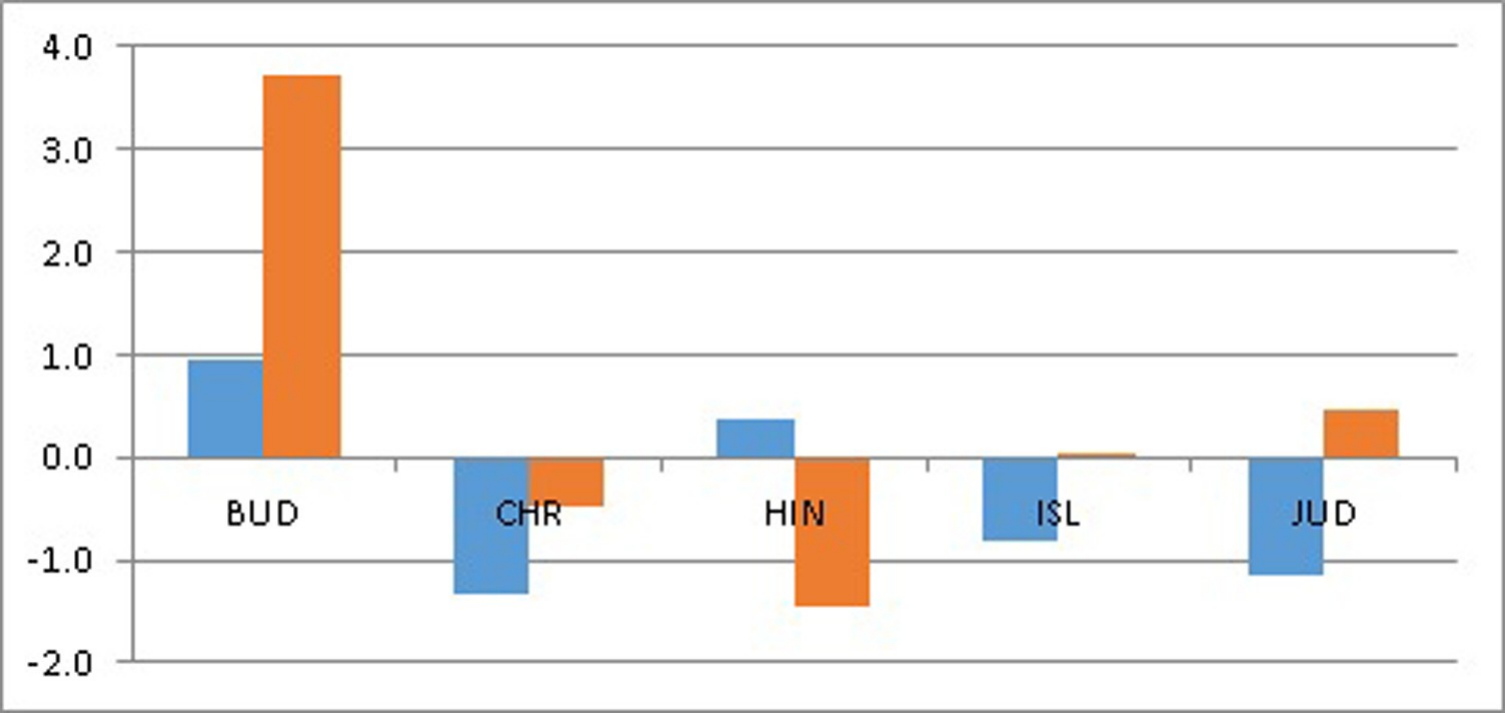
HLEB gains (positive values) and losses (negative values) in years from Buddhism, Christianity, Hinduism, Islam, and Judaism as majority religions (blue bars) and minority religions (red bars) within an each individual perspective.

Figs 2 and 3 show gains in health (years) and losses in happiness (0 to 10 scores) from religious choices within an *each* individual perspective based on *average* coefficients (i.e., Figs 2 and 3 do not depict the actual situation of each single person with regard to HLEB and LS). Let us refer to CHR as the paradigmatic religion since it positively affects LS and negatively affects HLEB, both as a majority and minority religion. Thus, BUD is peculiar, both as a majority and a minority religion, since it affects LS positively and HLEB negatively; HIN is similar to the paradigmatic religion, although it positively affects HLEB if it is a majority religion; ISL is also similar to the paradigmatic religion, although it does not affect either LS or HLEB if it is a minority religion; and JUD is also similar to the paradigmatic religion, although it positively affects HLEB if it is a minority religion.

Note that for both EDU and REL, each individual will personally combine these different units (i.e., combining these different units in percentages with respect to the initial status might be misleading). In summary, there is no *objectively* dominant choice of EDU levels or REL beliefs.

### Dynamics

The previous section showed that the dynamic interrelationships between happiness and health do not depend on contexts or policies (i.e., HLEB affects LS by approximately 0.086, whereas LSk4 affects HLEB by approximately 3.161). In this section, I use an average size of these estimated statistical coefficients (i.e., 0.086 and 3.161) within a dynamic theoretical model to specify whether and how the life model converges to the long-run equilibria of happiness and health. In particular, the two eigenvalues (i.e., -0.521 and 0.521) and the two eigenvectors (i.e., [-0.162, 0.986] and [0.162, 0.986]) based on these average coefficients suggest that the dynamic system is globally stable. Note that this result evokes “the enigma of health” by Gadamer [52]. Indeed, HLEB includes both the successful restoration of health due to medicine and the successful efforts of individuals to restore health thanks to nature (i.e., both medicine and nature). In addition, HLEB is used within a dynamic model where both health and happiness achieve a long-run equilibrium if shocks are not so frequent (i.e., re-establishing a dynamic rather than a static equilibrium) [53].

**Fig 4:**
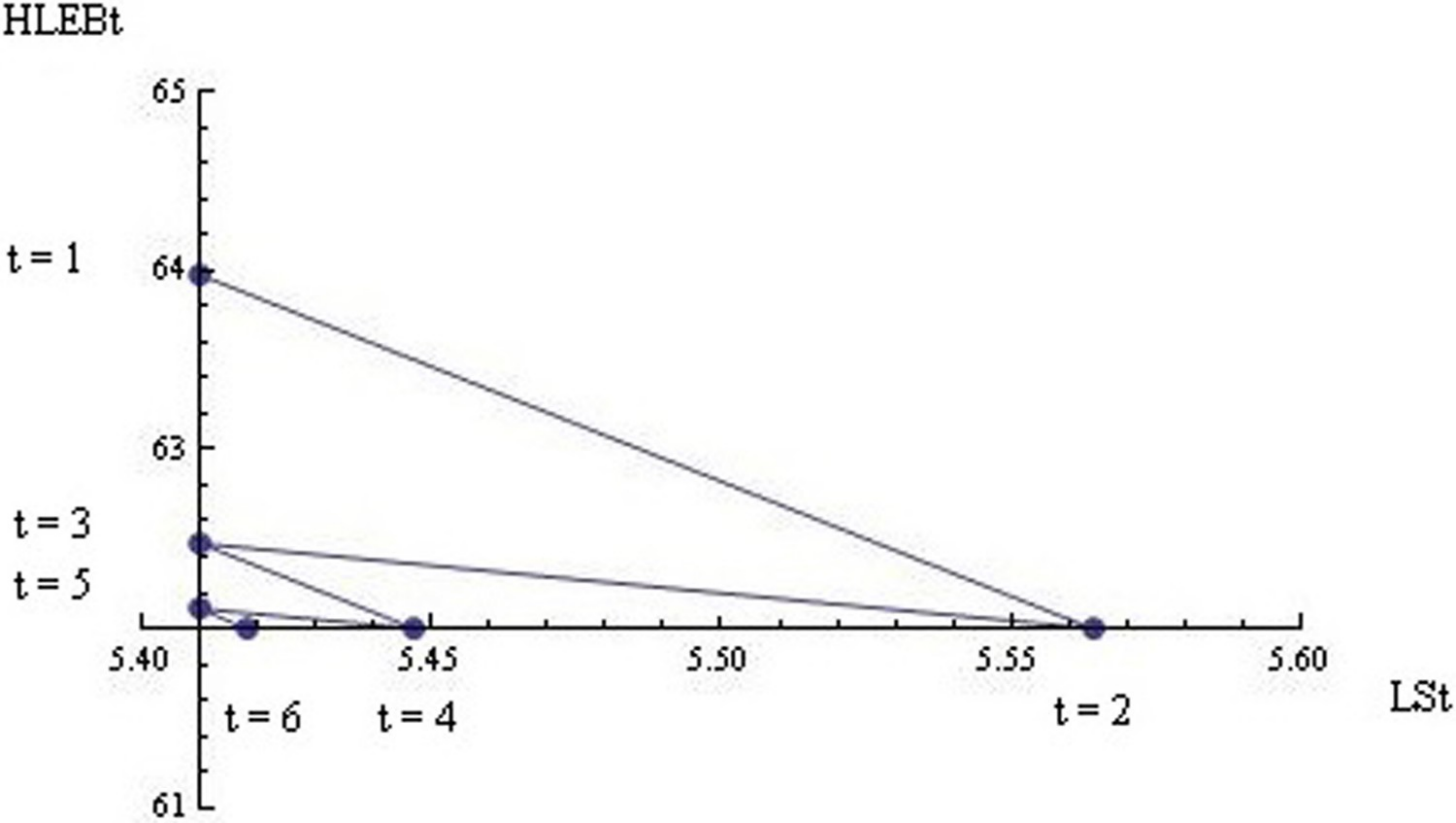
The oscillation dynamics of HLEB and LS towards the long-run equilibrium after a positive shock in LS within an intercultural representative individual perspective at the world level, if k is set at 1 and both constant of integrations are fixed at 0.

Fig 4 shows the oscillation dynamics of health and happiness towards the long-run equilibrium within an intercultural *representative* individual perspective at the world level. Thus, regardless of contexts, it takes 8 years for individuals to achieve the long-run equilibrium of happiness and health after a positive or negative shock on either happiness or health (i.e., unfortunate events that are negligible on average at the country level but that are crucial for each individual at a personal level), although the level of this equilibrium depends on the social and individual religions and social ethics prevailing in his or her country, as well as on the degree of development and inequality characterising his or her country.

Note that *surviving* individuals who do not manage to cope with their shocks are also included in these dynamics, although at low levels of health and happiness. In summary, there is a *single* life model for all individuals in the world.

## Discussion

Some methodological remarks are noteworthy here.

With a theoretical approach, Zagonari [54] shows that moral philosophy and theology can function as behavioural sciences. The applied approach adopted in this paper shows that the use of a representative individual at the country level is the most suitable perspective with which to analyse religious and secular ethics. Indeed, this approach enables the application of results from statistical estimations to characterise the dynamics of an analytical model representing both individual and social ethics.

In particular, two main strengths of the present study should be stressed as follows:

- I replicate the main literature insights about the effects of REL and EDU on happiness and health by detailing both REL and EDU. Indeed, the average of the significant coefficients obtained for the 5 main religions and the 3 education levels (i.e., links of average REL with happiness in Tables 3–6 are 0.52, 0.59, 0.21, 0.51, respectively; links of average REL with health in Tables 3–6 are -0.26, -0.22, 2.96, -1.31, respectively) suggest that REL is good for happiness, but it is bad for health, whereas EDU is good for health, but it is bad for happiness; however, religious precepts might be beneficial for health if secular social capital is well established. Nevertheless, both REL and EDU highlight some interdependencies (i.e., individual knowledge shows a larger positive link with happiness for more communitarian religions such as JUD and CHR, and social capital shows a more positive link with health for more individualistic religions such as BUD).
- I replicate the main literature insights about the effects of REL and EDU on happiness and health by using a more suitable measure of health. Indeed, the estimations based on LEB provided in S9-S12 Tables in S1 File are relatively similar to the estimations based on HLEB presented in the main text in terms of relative linkages of REL and EDU (i.e., Hindu and Muslim people are happier than atheists by considering LEB, while Buddhist, Hindu and Muslim people are healthier than atheists by considering HLEB, where the happiness and health gained from these religions are overestimated and underestimated, respectively). However, the use of HLEB instead of LEB explains LS to a greater extent (i.e., R^2^ at 0.61 instead of 0.58).

In contrast, two main weaknesses of the present study should be stressed as follows:

- There is a temporal mismatch between cohorts in dependent and independent variables: LEB and HLEB refer to children, EDU refers to adolescents, and LS and REL refer to adults. However, LEB and HLEB are updated with reference to adults (e.g., by referring to children, HLEB decreased in 2021 due to COVID-19), while adults are assumed to be over 14 years old (i.e., tertiary education refers to adults).
- The adopted representative individual perspective at the country level misses individual characteristics (e.g., gender, family status such as married/divorced/widowed/single, age, income, employment status such as full/part time, self/employees, housewife/student, risk behaviours such as tobacco, alcohol, drug uses); it also misses specific features of religiosity (e.g., its absolute and relative importance with respect to other ethics, beliefs, orientation, church attendance, daily hours of pray). However, the individual approach relies on self-reported health rather than on official health statistics at the national level. Moreover, the sample for variables at the country level is more representative than a large sample of interviews, and 162 countries represent almost all countries in the world. Finally, the individual approach misses the social impacts of REL ethics and EDU policies.

In summary, from a methodological perspective, the use of a representative individual at the country level can use most reliable data by relying on the minimum set of assumptions, although inequalities arising from religious and educational systems are disregarded (e.g., gender inequality in HLEB [55]; gender inequality in LS [56]).

In addition, from a practical perspective, together with individual choices related to education levels or religious beliefs to achieve higher levels of happiness and health in both the short-run and the long-run, some policy insights are worthy here, although three preliminary clarifications are needed.

First, the goal of this paper is not to provide policy implications but rather to evaluate the direct and indirect impacts of individual and social ethics from EDU and REL on health and happiness, as well as to specify the time lag and the significant size of the indirect impact of happiness on health. Consequently, the only variables on which to base policy insights are per capita income and income inequality since health and education expenditures per capita are introduced as control variables.

Second, this paper refers to the average individual in each country. Consequently, possible policy implications could refer to improving the *average* happiness and health levels in each country, although this approach might be unsatisfactory, since alternative approaches could be preferred. That is, equal opportunities for happiness (e.g., [57]), although happy or flourishing societies as a necessary condition for happy or flourishing citizens should be a goal of governments, as stated by Turner [58]; or equal access to health care (e.g., [59]), although health equity rather than equal health should be a goal of governments, as stated by Woolf [60].

Third, the positive and significant impact of per capita income in all contexts (i.e., REL majorities vs. percentages, EDU enrolment vs. expenditure) is a well-established result in the literature. Consequently, policy implications can be obtained only from the impacts of income inequality on average happiness and health.

For *happiness*, income inequality as measured by the Gini index could represent inequality in happiness opportunities. There are many cross-country papers that focus on the relationship between income inequality and average happiness levels. For example, the negative effect of income inequality on average happiness is shown to be larger in countries characterised by a larger proportion of poor people [61], worse public services [62], worse social mobility [63], worse civil liberties and political rights [64], worse job security policies [65] or worse work-life balance policies [66].

Thus, employment policies properly stratified in terms of gender and age seem to be the most popular policy suggestion to improve average happiness, together with interventions aiming to increase social mobility and reduce relative poverty.

For *health*, income inequality as measured by the Gini index could represent inequality in health-care access. There are many cross-country papers that focus on the relationship between income inequality and average health status. For example, the negative effect of income inequality on average health status is shown to be larger in countries characterised by market-based more than community-based health-care strategies [67], a larger range of age and gender inequality [68, 69], a larger range of relative poverty [70], a larger range of absolute poverty [71] or a less efficient health-care system [72].

Thus, health-care policies properly tailored in terms of gender and age seem to be the most popular policy suggestion to improve average health status, together with interventions that focus on housing, transportation, and public safety.

In summary, from a practical perspective, under the assumption that the Gini index well represents inequality both in happiness opportunities (i.e., a more subjective dimension) and in health-care access (i.e., a more objective dimension), policy-makers at the country level *could* face a clash with regard to reducing inequality with a positive impact on average health and increasing inequality with a positive impact on average happiness whenever the country is characterised by an average level of happiness that is larger than the median level of happiness. In other words, a similar clash between health and happiness can be observed both at the individual and the policy-maker level.

## Conclusion

The *first* purpose of this paper was to evaluate the direct and indirect impacts (and their interactions) of individual and social ethics from education and religion on health and happiness in alternative contexts and for alternative policies. The statistical results show that there is no religious or secular ethics with beneficial impacts on both happiness and health at both the individual and social levels. This insight is crucial to keep one from expecting the whole world population to adopt the same religious or secular ethics in the future. Next, education policies (gross enrolment and per-student expenditure) have similar impacts on both happiness and health in all religious contexts, while some religions have different impacts on happiness or health if coupled with different education policies. This insight suggests increasing both gross enrolment and per-student expenditure for secondary education in *all* countries, while the per-student expenditure for tertiary education might have negative impacts on happiness.

The *second* purpose of this study was to specify the time lag for the short-run indirect impact (and its size) of happiness on health and the (globally stable) long-run equilibria of both happiness and health within a statistical dynamic framework. Combined statistical and analytical results show that the largest short-run indirect impact of happiness on health occurs after 4 years, where 1 out of 10 points of happiness produces 3 additional years of healthy life expectancy at birth. This insight requires each individual to personally evaluate gains in happiness *in terms of* losses in health. Next, the long-run equilibria of both happiness and health are globally stable, and they are achieved after 8 years through oscillation dynamics. This insight is essential to explaining the past distribution of individuals’ happiness and health levels across countries, although these levels *depend on* income and inequality.

In summary, both religious and secular ethics are needed to achieve both happiness and health for two main reasons. First, there is a static beneficial interaction (i.e., for each time) between religious and secular ethics. In particular, majority religions mainly show beneficial linkages with happiness by providing social capital, although quantity of education could offset a reduction in believer proportion, in particular where more communitarian religions prevail (e.g., Christianity and Judaism). Similarly, quality of education mainly shows beneficial linkages with health by providing individual knowledge, although quantity of education could offset a reduction in quality of education, in particular where more individualistic religions prevail (e.g., Buddhism). Second, there is a dynamic beneficial interaction (i.e., across times) between religious and secular ethics. In particular, an increase in happiness produces an increase in health with a time lag. Similarly, an increase in health produces an increase in happiness with no time lag.

Note that there is no dominant choice of secular vs. religious ethics within an each individual perspective (i.e., there is no single optimal balance between health and happiness for anybody). Moreover, education policies should be pursued in any religious context within a representative individual perspective (i.e., linkages of education policies do not depend on religious contexts, whereas linkages of social and individual religious ethics might depend on education policies). Finally, the previous insights about static and dynamic interactions between religious and secular ethics seem to be empirically supported by the observed dynamics of education achievements, religious believers and life satisfaction in OECD countries from 2000 to 2020. Indeed, consistently with the theoretical insight by Schopenhauer (i.e., religions represent metaphysics of people and an increase in tertiary education within a cultural individual perspective *cannot* compensate for a decrease in believer proportion in providing a meaning to life) [73]: education quantity in primary, secondary and tertiary increased by 114%, 107%, and 143%, respectively (by reaching *the practically highest possible* 100%, 123% and 102% of gross enrolment rates, respectively, in 2020); education quality in primary, secondary and tertiary increased by 115%, 93%, and 96%, respectively (by reaching 5221, 5199 and 7022 USD per-student per-year education expenditures, respectively, in 2020); believer proportion decreased by 5% (by falling to 78% in 2020); HLEB increased by 7% (by rising to 72.32 years in 2020); and LS decreased by 1% (by falling to 6.74 in 2020) [74].

The present study could be developed by considering specific groups of individuals (e.g., young vs. old people to evaluate the different impacts of secularisation; male vs. female to evaluate the different impacts of education policies). However, the specific statistical results (i.e., different parameter values and significances) are unlikely to *qualitatively* challenge the general analytical insight on the global stability of the life model. Moreover, additional religions could be included. However, the main religions used in the present paper (i.e., BUD, CHR, HIN, ISL, JUD) account for a *large* proportion (i.e., 87%) of the world population. Finally, variables across countries could be weighted according to the world population percentages by referring to a world average representative individual rather than to a world intercultural representative individual. However, some religions (e.g., BUD and JUD) would likely become *quantitatively* irrelevant.

In addition, some indirect insights (i.e., inconclusive statistical significance in certain predictors) obtained by the present study underscore the need for an empirical literature with important practical implications. In particular, social capital from minority religions and social capital from quality of education turned out to be indecisive in fostering ethics for happiness (i.e., they are unreliable predictors to increase happiness). These indirect results seem to suggest a higher emphasis on rights of believers in minority religions (e.g., economic and non-economic policies to favour mutual respect, peaceful relationships, and fair treatment of religions–in short, religious tolerance, by maintaining that your own religion is the only true one, whilst acknowledging other religions) [75]. Simultaneously, a deeper focus on education for duties seems to be warranted (e.g., economic and non-economic policies to increase responsibility, solidarity, and awareness of duties towards other people–in short, global citizenship, by stressing that your rights are important, however recognising cultural diversities) [76].

## Supporting information

S1 File Supporting materials containing S1-S24 Tables. (DOCX)
