# Supplementary material for: Both religious and secular ethics to achieve both happiness and health: Panel data results based on a dynamic theoretical model
Source: PLoS One. 2024 Apr 17;19(4):e0301905. doi: 10.1371/journal.pone.0301905 (PMC11023590; doi:10.1371/journal.pone.0301905)
Supplement: S1 File — (DOCX) [file pone.0301905.s001.docx]

# Supplementary Materials

**Table 1S. Levin-Lin-Chu unit-root test for LS. ADF regressions: 1 lag; LR variance: Bartlett kernel, 8.00 lags average (chosen by LLC).**

|  | Statistic | p-value |
| --- | --- | --- |
| Unadjusted t | -26.6209 |  |
| Adjusted t* | -5.3251 | 0.0000 |

**Table 2S. Levin-Lin-Chu unit-root test for HLEB. ADF regressions: 1 lag; LR variance: Bartlett kernel, 8.00 lags average (chosen by LLC).**

|  | Statistic | p-value |
| --- | --- | --- |
| Unadjusted t | -24.3600 |  |
| Adjusted t* | -9.7146 | 0.0000 |

**Table 3S. Dumitrescu and Hurlin test for Granger non-causality with a lag order at 1. H0: LS does not Granger-cause HLEB; H1: LS does Granger-cause HLEB for at least one panel.**

|  |  | P value |
| --- | --- | --- |
| W-bar | 5.1112 | 0.000 |
| Z-bar | 36.4250 | 0.000 |
| Z-bar tilde | 28.0308 | 0.000 |

**Table 4S. Dumitrescu and Hurlin test for Granger non-causality with a lag order at 1. H0: HLEB does not Granger-cause LS; H1: HLEB does Granger-cause LS for at least one panel.**

|  |  | P value |
| --- | --- | --- |
| W-bar | 3.2509 | 0.000 |
| Z-bar | 19.9434 | 0.000 |
| Z-bar tilde | 14.9223 | 0.000 |

**Table 5S. RELs & EDUs. No. of observations = 3240. R^2^ (ls) = 0.63, R^2^ (hleb) = 0.74.**

|  |  | Coef. | Std. Err. | z | P>z | [95% Conf. Interval] | |
| --- | --- | --- | --- | --- | --- | --- | --- |
| LS |  |  |  |  |  |  |  |
|  | lnGDP | .3621666 | .0196228 | 18.46 | 0.000 | .3237067 | .4006265 |
|  | GINI | .0021772 | .001839 | 1.18 | 0.236 | -.0014272 | .0057817 |
|  | **HLEBt-1** | .0683579 | .0027366 | 24.98 | 0.000 | .0629943 | .0737215 |
|  | BUDM | -.0393606 | .0662239 | -0.59 | 0.552 | -.169157 | .0904357 |
|  | CHRM | .4220646 | .0429521 | 9.83 | 0.000 | .3378801 | .5062492 |
|  | HINM | .2503313 | .1147419 | 2.18 | 0.029 | .0254413 | .4752213 |
|  | ISLM | .0605858 | .0458569 | 1.32 | 0.186 | -.0292921 | .1504637 |
|  | JUDM | 1.006761 | .1556856 | 6.47 | 0.000 | .7016226 | 1.311899 |
|  | GEP | -.0098856 | .0009149 | -10.80 | 0.000 | -.0116788 | -.0080924 |
|  | GES | .0016496 | .0008598 | 1.92 | 0.055 | -.0000356 | .0033348 |
|  | GET | -.004946 | .000737 | -6.71 | 0.000 | -.0063905 | -.0035015 |
|  | CONS | -1.380319 | .1876254 | -7.36 | 0.000 | -1.748058 | -1.01258 |
| HLEB |  |  |  |  |  |  |  |
|  | lnGDP | 1.358657 | .1255843 | 10.82 | 0.000 | 1.112517 | 1.604798 |
|  | GINI | -.0984011 | .010943 | -8.99 | 0.000 | -.119849 | -.0769531 |
|  | LSk4 | 2.837748 | .1157398 | 24.52 | 0.000 | 2.610902 | 3.064594 |
|  | BUDM | 1.328298 | .3980885 | 3.34 | 0.001 | .5480587 | 2.108537 |
|  | CHRM | -1.48238 | .2623771 | -5.65 | 0.000 | -1.99663 | -.9681305 |
|  | HINM | -.0851165 | .6918546 | -0.12 | 0.902 | -1.441127 | 1.270894 |
|  | ISLM | -.6240083 | .2761193 | -2.26 | 0.024 | -1.165192 | -.0828244 |
|  | JUDM | -.161197 | .9462604 | -0.17 | 0.865 | -2.015833 | 1.693439 |
|  | GEP | .0551247 | .005551 | 9.93 | 0.000 | .044245 | .0660045 |
|  | GES | .0527573 | .0050945 | 10.36 | 0.000 | .0427722 | .0627424 |
|  | GET | .052776 | .0043651 | 12.09 | 0.000 | .0442205 | .0613314 |
|  | CONS | 27.10878 | 1.034617 | 26.20 | 0.000 | 25.08097 | 29.13659 |

**Table 6S. RELi & EDUi. No. of observations = 3240. R^2^ (ls) = 0.62, R^2^ (hleb) = 0.73.**

|  |  | Coef. | Std. Err. | z | P>z | [95% Conf. Interval] | |
| --- | --- | --- | --- | --- | --- | --- | --- |
| LS |  |  |  |  |  |  |  |
|  | lnGDP | .1331854 | .023182 | 5.75 | 0.000 | .0877494 | .1786213 |
|  | GINI | .0093339 | .0019164 | 4.87 | 0.000 | .0055778 | .01309 |
|  | **HLEBt-1** | .0694525 | .0026881 | 25.84 | 0.000 | .0641839 | .0747211 |
|  | BUDP | -.0359186 | .1205412 | -0.30 | 0.766 | -.272175 | .2003378 |
|  | CHRP | .4131415 | .084976 | 4.86 | 0.000 | .2465916 | .5796914 |
|  | HINP | .6264295 | .1487347 | 4.21 | 0.000 | .3349149 | .917944 |
|  | ISLP | .172333 | .0805472 | 2.14 | 0.032 | .0144634 | .3302026 |
|  | JUDP | 1.255252 | .2209094 | 5.68 | 0.000 | .8222773 | 1.688226 |
|  | EEP | .0144175 | .0115433 | 1.25 | 0.212 | -.0082069 | .0370419 |
|  | EES | .0541818 | .0106895 | 5.07 | 0.000 | .0332307 | .0751329 |
|  | EET | .0011955 | .0014843 | 0.81 | 0.421 | -.0017137 | .0041046 |
|  | CONS | -.9640167 | .1783471 | -5.41 | 0.000 | -1.313571 | -.6144628 |
| HLEB |  |  |  |  |  |  |  |
|  | lnGDP | 4.111219 | .122017 | 33.69 | 0.000 | 3.87207 | 4.350368 |
|  | GINI | -.1443835 | .0114927 | -12.56 | 0.000 | -.1669088 | -.1218582 |
|  | LSk4 | 2.989974 | .1171836 | 25.52 | 0.000 | 2.760298 | 3.219649 |
|  | BUDP | 2.450742 | .7356625 | 3.33 | 0.001 | 1.00887 | 3.892615 |
|  | CHRP | -.7336238 | .5222046 | -1.40 | 0.160 | -1.757126 | .2898784 |
|  | HINP | -2.205953 | .912545 | -2.42 | 0.016 | -3.994508 | -.4173973 |
|  | ISLP | -.9889038 | .4929066 | -2.01 | 0.045 | -1.954983 | -.0228247 |
|  | JUDP | -.1326394 | 1.360887 | -0.10 | 0.922 | -2.799929 | 2.53465 |
|  | EEP | .3583307 | .0702753 | 5.10 | 0.000 | .2205937 | .4960678 |
|  | EES | -.460649 | .0651091 | -7.08 | 0.000 | -.5882606 | -.3330374 |
|  | EET | -.1242429 | .0087791 | -14.15 | 0.000 | -.1414496 | -.1070362 |
|  | CONS | 15.79264 | 1.065116 | 14.83 | 0.000 | 13.70505 | 17.88022 |

**Table 7S. RELi & EDUs. No. of observations = 3240. R^2^ (ls) = 0.62, R^2^ (hleb) = 0.75.**

|  |  | Coef. | Std. Err. | z | P>z | [95% Conf. Interval] | |
| --- | --- | --- | --- | --- | --- | --- | --- |
| LS |  |  |  |  |  |  |  |
|  | lnGDP | .3615389 | .0197259 | 18.33 | 0.000 | .3228769 | .400201 |
|  | GINI | .0028224 | .0018681 | 1.51 | 0.131 | -.0008391 | .0064839 |
|  | **HLEBt-1** | .0680326 | .0027794 | 24.48 | 0.000 | .062585 | .0734802 |
|  | BUDP | -.319628 | .1208441 | -2.64 | 0.008 | -.556478 | -.082778 |
|  | CHRP | .2964288 | .0849198 | 3.49 | 0.000 | .1299892 | .4628685 |
|  | HINP | .3430303 | .1515247 | 2.26 | 0.024 | .0460473 | .6400133 |
|  | ISLP | -.1532215 | .0801521 | -1.91 | 0.056 | -.3103167 | .0038736 |
|  | JUDP | 1.125444 | .2193664 | 5.13 | 0.000 | .6954934 | 1.555394 |
|  | GEP | -.0102067 | .0009174 | -11.13 | 0.000 | -.0120047 | -.0084087 |
|  | GES | .0018751 | .0008657 | 2.17 | 0.030 | .0001785 | .0035718 |
|  | GET | -.0049499 | .0007603 | -6.51 | 0.000 | -.00644 | -.0034598 |
|  | CONS | -1.208384 | .1975478 | -6.12 | 0.000 | -1.595571 | -.821198 |
| HLEB |  |  |  |  |  |  |  |
|  | lnGDP | 1.415675 | .1239437 | 11.42 | 0.000 | 1.17275 | 1.6586 |
|  | GINI | -.1039307 | .0109529 | -9.49 | 0.000 | -.1253981 | -.0824634 |
|  | LSk4 | 2.737691 | .1138586 | 24.04 | 0.000 | 2.514532 | 2.96085 |
|  | BUDP | 4.879886 | .7127608 | 6.85 | 0.000 | 3.482901 | 6.276872 |
|  | CHRP | -.0413883 | .5057337 | -0.08 | 0.935 | -1.032608 | .9498315 |
|  | HINP | -.6488177 | .9011481 | -0.72 | 0.472 | -2.415035 | 1.1174 |
|  | ISLP | .8656021 | .4758257 | 1.82 | 0.069 | -.0669992 | 1.798203 |
|  | JUDP | 2.014199 | 1.310235 | 1.54 | 0.124 | -.553815 | 4.582214 |
|  | GEP | .0556805 | .0054914 | 10.14 | 0.000 | .0449176 | .0664435 |
|  | GES | .0520293 | .0050621 | 10.28 | 0.000 | .0421078 | .0619509 |
|  | GET | .0530208 | .0044398 | 11.94 | 0.000 | .044319 | .0617227 |
|  | CONS | 25.94605 | 1.089616 | 23.81 | 0.000 | 23.81044 | 28.08165 |

**Table 8S. RELs & EDUi. No. of observations = 3240. R^2^ (ls) = 0.63, R^2^ (hleb) = 0.73.**

|  |  | Coef. | Std. Err. | z | P>z | [95% Conf. Interval] | |
| --- | --- | --- | --- | --- | --- | --- | --- |
| LS |  |  |  |  |  |  |  |
|  | lnGDP | .1418007 | .023043 | 6.15 | 0.000 | .0966373 | .1869641 |
|  | GINI | .0082829 | .0018956 | 4.37 | 0.000 | .0045675 | .0119982 |
|  | **HLEBt-1** | .0689701 | .0026617 | 25.91 | 0.000 | .0637532 | .0741869 |
|  | BUDM | .0476696 | .0660127 | 0.72 | 0.470 | -.0817129 | .1770521 |
|  | CHRM | .3805948 | .0426956 | 8.91 | 0.000 | .2969129 | .4642766 |
|  | HINM | .1556135 | .1133174 | 1.37 | 0.170 | -.0664844 | .3777115 |
|  | ISLM | .2006994 | .0462733 | 4.34 | 0.000 | .1100055 | .2913934 |
|  | JUDM | 1.035629 | .1571324 | 6.59 | 0.000 | .7276547 | 1.343602 |
|  | EEP | .0056149 | .0114935 | 0.49 | 0.625 | -.0169119 | .0281417 |
|  | EES | .0571127 | .0106718 | 5.35 | 0.000 | .0361963 | .078029 |
|  | EET | .0011443 | .0014731 | 0.78 | 0.437 | -.0017429 | .0040316 |
|  | CONS | -.9580514 | .1690584 | -5.67 | 0.000 | -1.2894 | -.626703 |
| HLEB |  |  |  |  |  |  |  |
|  | lnGDP | 4.115608 | .1230033 | 33.46 | 0.000 | 3.874526 | 4.35669 |
|  | GINI | -.1377943 | .0114931 | -11.99 | 0.000 | -.1603204 | -.1152682 |
|  | LSk4 | 3.032863 | .1187658 | 25.54 | 0.000 | 2.800087 | 3.26564 |
|  | BUDM | .4992976 | .4076455 | 1.22 | 0.221 | -.2996728 | 1.298268 |
|  | CHRM | -.9624704 | .267727 | -3.59 | 0.000 | -1.487206 | -.437735 |
|  | HINM | .8253434 | .7002007 | 1.18 | 0.239 | -.5470248 | 2.197712 |
|  | ISLM | -1.108782 | .2865289 | -3.87 | 0.000 | -1.670368 | -.5471956 |
|  | JUDM | -1.390596 | .9796192 | -1.42 | 0.156 | -3.310614 | .5294228 |
|  | EEP | .4066077 | .0705626 | 5.76 | 0.000 | .2683075 | .5449079 |
|  | EES | -.4905216 | .0656285 | -7.47 | 0.000 | -.6191512 | -.3618921 |
|  | EET | -.126979 | .0087899 | -14.45 | 0.000 | -.1442069 | -.1097512 |
|  | CONS | 15.47676 | 1.018513 | 15.20 | 0.000 | 13.48051 | 17.473 |

**Table 9S. Impact of LEB on RELs & EDUs. R^2^ (ls) = 0.58, R^2^ (hleb) = 0.74.**

|  |  | Coef. | Std. Err. | z | P>z | [95% Conf. | Interval] |
| --- | --- | --- | --- | --- | --- | --- | --- |
| LS |  |  |  |  |  |  |  |
|  | lnGDP | .3152124 | .0203519 | 15.49 | 0.000 | .2753234 | .3551014 |
|  | GINI | .0041748 | .0018853 | 2.21 | 0.027 | .0004796 | .00787 |
|  | **LEB** | .0700354 | .0026775 | 26.16 | 0.000 | .0647876 | .0752832 |
|  | BUDM | -.1266016 | .0677883 | -1.87 | 0.062 | -.2594643 | .0062611 |
|  | CHRM | .3757901 | .0436644 | 8.61 | 0.000 | .2902095 | .4613707 |
|  | HINM | .097649 | .1175821 | 0.83 | 0.406 | -.1328077 | .3281057 |
|  | ISLM | -.04595 | .0466598 | -0.98 | 0.325 | -.1374016 | .0455015 |
|  | JUDM | .9183191 | .1595107 | 5.76 | 0.000 | .6056839 | 1.230954 |
|  | GEP | -.0100133 | .0009257 | -10.82 | 0.000 | -.0118277 | -.0081989 |
|  | GES | .0009545 | .0008821 | 1.08 | 0.279 | -.0007743 | .0026834 |
|  | GET | -.0053044 | .0007552 | -7.02 | 0.000 | -.0067846 | -.0038243 |
|  | CONS | -1.571392 | .1918105 | -8.19 | 0.000 | -1.947334 | -1.195451 |
| **LEB** |  |  |  |  |  |  |  |
|  | lnGDP | 1.67401 | .1381306 | 12.12 | 0.000 | 1.403279 | 1.944741 |
|  | GINI | -.1239293 | .0121515 | -10.20 | 0.000 | -.1477457 | -.1001128 |
|  | LSk4 | 3.186843 | .125968 | 25.30 | 0.000 | 2.93995 | 3.433735 |
|  | BUDM | 2.252586 | .4427361 | 5.09 | 0.000 | 1.384839 | 3.120333 |
|  | CHRM | -1.282637 | .2902812 | -4.42 | 0.000 | -1.851578 | -.7136963 |
|  | HINM | 1.630404 | .7709443 | 2.11 | 0.034 | .1193809 | 3.141427 |
|  | ISLM | .7445326 | .3057262 | 2.44 | 0.015 | .1453202 | 1.343745 |
|  | JUDM | .2197013 | 1.054397 | 0.21 | 0.835 | -1.846878 | 2.286281 |
|  | GEP | .0598109 | .006108 | 9.79 | 0.000 | .0478394 | .0717824 |
|  | GES | .0652311 | .0056477 | 11.55 | 0.000 | .0541619 | .0763004 |
|  | GET | .0538138 | .0048806 | 11.03 | 0.000 | .044248 | .0633797 |
|  | CONS | 29.46492 | 1.136332 | 25.93 | 0.000 | 27.23775 | 31.69209 |

**Table 10S. Impact of LEB on RELi & EDUi. R^2^ (ls) = 0.58, R^2^ (hleb) = 0.72.**

|  |  | Coef. | Std. Err. | z | P>z | [95% Conf. | Interval] |
| --- | --- | --- | --- | --- | --- | --- | --- |
| LS |  |  |  |  |  |  |  |
|  | lnGDP | .0397895 | .0247492 | 1.61 | 0.108 | -.0087181 | .0882971 |
|  | GINI | .0125149 | .0019653 | 6.37 | 0.000 | .0086629 | .0163669 |
|  | **LEB** | .0733658 | .0026146 | 28.06 | 0.000 | .0682413 | .0784904 |
|  | BUDP | -.1648716 | .1210985 | -1.36 | 0.173 | -.4022202 | .072477 |
|  | CHRP | .3194926 | .0844612 | 3.78 | 0.000 | .1539516 | .4850335 |
|  | HINP | .3778622 | .1509039 | 2.50 | 0.012 | .0820959 | .6736284 |
|  | ISLP | .0336128 | .0801189 | 0.42 | 0.675 | -.1234174 | .190643 |
|  | JUDP | 1.11318 | .2256866 | 4.93 | 0.000 | .6708428 | 1.555518 |
|  | EEP | .000222 | .0120294 | 0.02 | 0.985 | -.0233553 | .0237992 |
|  | EES | .0706455 | .0111235 | 6.35 | 0.000 | .0488437 | .0924472 |
|  | EET | .0016405 | .0014886 | 1.10 | 0.270 | -.001277 | .004558 |
|  | _CONS | -1.004483 | .1813701 | -5.54 | 0.000 | -1.359962 | -.6490042 |
| **LEB** |  |  |  |  |  |  |  |
|  | lnGDP | 4.879405 | .1349637 | 36.15 | 0.000 | 4.614881 | 5.143929 |
|  | GINI | -.1875305 | .0127617 | -14.69 | 0.000 | -.212543 | -.162518 |
|  | LSk4 | 3.471811 | .1275022 | 27.23 | 0.000 | 3.221912 | 3.721711 |
|  | BUDP | 3.803403 | .8070017 | 4.71 | 0.000 | 2.221708 | 5.385097 |
|  | CHRP | -.0033518 | .5674597 | -0.01 | 0.995 | -1.115552 | 1.108849 |
|  | HINP | .6303124 | 1.011763 | 0.62 | 0.533 | -1.352707 | 2.613332 |
|  | ISLP | .7142674 | .5360955 | 1.33 | 0.183 | -.3364604 | 1.764995 |
|  | JUDP | .5100458 | 1.519397 | 0.34 | 0.737 | -2.467918 | 3.48801 |
|  | EEP | .4902555 | .0799095 | 6.14 | 0.000 | .3336358 | .6468752 |
|  | EES | -.69582 | .0738072 | -9.43 | 0.000 | -.8404793 | -.5511606 |
|  | EET | -.1179611 | .0096809 | -12.18 | 0.000 | -.1369353 | -.0989869 |
|  | CONS | 15.4356 | 1.18413 | 13.04 | 0.000 | 13.11475 | 17.75645 |

**Table 11S. Impact of LEB on RELi & EDUs. R^2^ (ls) = 0.58, R^2^ (hleb) = 0.74.**

|  |  | Coef. | Std. Err. | z | P>z | [95% Conf. | Interval] |
| --- | --- | --- | --- | --- | --- | --- | --- |
| LS |  |  |  |  |  |  |  |
|  | lnGDP | .3142862 | .0204123 | 15.40 | 0.000 | .2742789 | .3542935 |
|  | GINI | .0047138 | .0019104 | 2.47 | 0.014 | .0009696 | .0084581 |
|  | **LEB** | .0702125 | .0027168 | 25.84 | 0.000 | .0648877 | .0755373 |
|  | BUDP | -.4078992 | .1218184 | -3.35 | 0.001 | -.6466588 | -.1691395 |
|  | CHRP | .2504458 | .0845653 | 2.96 | 0.003 | .0847009 | .4161908 |
|  | HINP | .1330961 | .1541684 | 0.86 | 0.388 | -.1690685 | .4352606 |
|  | ISLP | -.269191 | .0800515 | -3.36 | 0.001 | -.426089 | -.1122931 |
|  | JUDP | 1.005742 | .2239379 | 4.49 | 0.000 | .5668318 | 1.444652 |
|  | GEP | -.0104069 | .000927 | -11.23 | 0.000 | -.0122238 | -.00859 |
|  | GES | .0011369 | .0008863 | 1.28 | 0.200 | -.0006002 | .002874 |
|  | GET | -.0054741 | .0007781 | -7.04 | 0.000 | -.0069992 | -.003949 |
|  | CONS | -1.405404 | .2002156 | -7.02 | 0.000 | -1.797819 | -1.012988 |
| **LEB** |  |  |  |  |  |  |  |
|  | lnGDP | 1.709264 | .136298 | 12.54 | 0.000 | 1.442124 | 1.976403 |
|  | GINI | -.1274803 | .0121528 | -10.49 | 0.000 | -.1512995 | -.1036612 |
|  | LSk4 | 3.092713 | .1239337 | 24.95 | 0.000 | 2.849808 | 3.335619 |
|  | BUDP | 6.412656 | .7804154 | 8.22 | 0.000 | 4.88307 | 7.942242 |
|  | CHRP | .5881425 | .5490789 | 1.07 | 0.284 | -.4880324 | 1.664317 |
|  | HINP | 2.346079 | .9981425 | 2.35 | 0.019 | .3897555 | 4.302402 |
|  | ISLP | 2.844454 | .5163644 | 5.51 | 0.000 | 1.832398 | 3.856509 |
|  | JUDP | 2.99997 | 1.45667 | 2.06 | 0.039 | .1449495 | 5.854991 |
|  | GEP | .0608479 | .0060432 | 10.07 | 0.000 | .0490035 | .0726924 |
|  | GES | .0641972 | .0056078 | 11.45 | 0.000 | .0532061 | .0751883 |
|  | GET | .0564597 | .0049575 | 11.39 | 0.000 | .0467431 | .0661764 |
|  | CONS | 27.81507 | 1.191255 | 23.35 | 0.000 | 25.48026 | 30.14989 |

**Table 12S. Impact of LEB on RELs & EDUi. R^2^ (ls) = 0.58, R^2^ (hleb) = 0.72.**

|  |  | Coef. | Std. Err. | z | P>z | [95% Conf. | Interval] |
| --- | --- | --- | --- | --- | --- | --- | --- |
| LS |  |  |  |  |  |  |  |
|  | lnGDP | .0469027 | .0246967 | 1.90 | 0.058 | -.0015019 | .0953074 |
|  | GINI | .011532 | .0019462 | 5.93 | 0.000 | .0077176 | .0153465 |
|  | **LEB** | .072898 | .0025967 | 28.07 | 0.000 | .0678084 | .0779875 |
|  | BUDM | -.0414755 | .0674902 | -0.61 | 0.539 | -.1737537 | .0908028 |
|  | CHRM | .3214541 | .043469 | 7.40 | 0.000 | .2362565 | .4066517 |
|  | HINM | -.0125362 | .1162548 | -0.11 | 0.914 | -.2403915 | .2153191 |
|  | ISLM | .0962937 | .0470253 | 2.05 | 0.041 | .0041259 | .1884615 |
|  | JUDM | .9555899 | .1610135 | 5.93 | 0.000 | .6400093 | 1.271171 |
|  | EEP | -.0058005 | .0119777 | -0.48 | 0.628 | -.0292764 | .0176755 |
|  | EES | .0722193 | .0111073 | 6.50 | 0.000 | .0504493 | .0939892 |
|  | EET | .0015615 | .0014789 | 1.06 | 0.291 | -.001337 | .00446 |
|  | CONS | -1.017316 | .1723163 | -5.90 | 0.000 | -1.355049 | -.6795822 |
| **LEB** |  |  |  |  |  |  |  |
|  | lnGDP | 4.89727 | .1358728 | 36.04 | 0.000 | 4.630964 | 5.163576 |
|  | GINI | -.1819083 | .0127349 | -14.28 | 0.000 | -.2068683 | -.1569484 |
|  | LSk4 | 3.510809 | .1289552 | 27.23 | 0.000 | 3.258062 | 3.763557 |
|  | BUDM | 1.298901 | .4541198 | 2.86 | 0.004 | .4088422 | 2.188959 |
|  | CHRM | -.6703224 | .2967363 | -2.26 | 0.024 | -1.251915 | -.0887301 |
|  | HINM | 2.546064 | .7818628 | 3.26 | 0.001 | 1.013641 | 4.078487 |
|  | ISLM | .0615116 | .317434 | 0.19 | 0.846 | -.5606476 | .6836708 |
|  | JUDM | -1.335934 | 1.093901 | -1.22 | 0.222 | -3.479942 | .808073 |
|  | EEP | .5157362 | .0800657 | 6.44 | 0.000 | .3588104 | .6726621 |
|  | EES | -.7150646 | .0741919 | -9.64 | 0.000 | -.8604781 | -.5696512 |
|  | EET | -.1198331 | .0096767 | -12.38 | 0.000 | -.1387992 | -.1008671 |
|  | _CONS | 15.51558 | 1.129973 | 13.73 | 0.000 | 13.30087 | 17.73029 |

**Table 13S. Impact of k at 3 on RELs & EDUs. R^2^ (ls) = 0.61, R^2^ (hleb) = 0.71.**

|  |  | Coef. | Std. Err. | z | P>z | [95% Conf. | Interval] |
| --- | --- | --- | --- | --- | --- | --- | --- |
| LS |  |  |  |  |  |  |  |
|  | lnGDP | .3244021 | .0197 | 16.47 | 0.000 | .2857909 | .3630133 |
|  | GINI | .0036226 | .001837 | 1.97 | 0.049 | .0000223 | .007223 |
|  | HLEB | .0799375 | .0029685 | 26.93 | 0.000 | .0741195 | .0857556 |
|  | BUDM | -.0745387 | .0660398 | -1.13 | 0.259 | -.2039743 | .0548969 |
|  | CHRM | .4121503 | .0426664 | 9.66 | 0.000 | .3285256 | .495775 |
|  | HINM | .2226476 | .1146298 | 1.94 | 0.052 | -.0020227 | .4473178 |
|  | ISLM | .0524985 | .0455269 | 1.15 | 0.249 | -.0367326 | .1417296 |
|  | JUDM | .9425294 | .1557003 | 6.05 | 0.000 | .6373625 | 1.247696 |
|  | GEP | -.0104166 | .0009056 | -11.50 | 0.000 | -.0121915 | -.0086417 |
|  | GES | .0010535 | .0008601 | 1.22 | 0.221 | -.0006322 | .0027392 |
|  | GET | -.005561 | .000739 | -7.52 | 0.000 | -.0070095 | -.0041126 |
|  | CONS | -1.673053 | .188771 | -8.86 | 0.000 | -2.043037 | -1.303068 |
| HLEB |  |  |  |  |  |  |  |
|  | lnGDP | 1.125757 | .1312542 | 8.58 | 0.000 | .8685036 | 1.383011 |
|  | GINI | -.0982952 | .0115014 | -8.55 | 0.000 | -.1208375 | -.0757529 |
|  | **LSk3** | 3.194746 | .1210148 | 26.40 | 0.000 | 2.957562 | 3.431931 |
|  | BUDM | 1.307297 | .4189464 | 3.12 | 0.002 | .4861776 | 2.128417 |
|  | CHRM | -1.725206 | .2747673 | -6.28 | 0.000 | -2.26374 | -1.186672 |
|  | HINM | -.2150583 | .7294497 | -0.29 | 0.768 | -1.644753 | 1.214637 |
|  | ISLM | -.5678908 | .2892836 | -1.96 | 0.050 | -1.134876 | -.0009054 |
|  | JUDM | -.5936805 | .9981003 | -0.59 | 0.552 | -2.549921 | 1.36256 |
|  | GEP | .0604947 | .0057824 | 10.46 | 0.000 | .0491613 | .0718281 |
|  | GES | .0531302 | .0053459 | 9.94 | 0.000 | .0426524 | .0636079 |
|  | GET | .0512862 | .004619 | 11.10 | 0.000 | .0422332 | .0603392 |
|  | CONS | 26.78788 | 1.075545 | 24.91 | 0.000 | 24.67985 | 28.89591 |

**Table 14S. Impact of k at 3 on RELi & EDUi. R^2^ (ls) = 0.62, R^2^ (hleb) = 0.70.**

|  |  | Coef. | Std. Err. | z | P>z | [95% Conf. | Interval] |
| --- | --- | --- | --- | --- | --- | --- | --- |
| LS |  |  |  |  |  |  |  |
|  | lnGDP | .0793771 | .0238052 | 3.33 | 0.001 | .0327199 | .1260344 |
|  | GINI | .0106187 | .0019119 | 5.55 | 0.000 | .0068714 | .0143659 |
|  | HLEB | .0796605 | .0029183 | 27.30 | 0.000 | .0739408 | .0853802 |
|  | BUDP | -.0932653 | .1182785 | -0.79 | 0.430 | -.3250869 | .1385563 |
|  | CHRP | .3793729 | .082547 | 4.60 | 0.000 | .2175838 | .5411621 |
|  | HINP | .5910012 | .1474417 | 4.01 | 0.000 | .3020208 | .8799816 |
|  | ISLP | .1542392 | .0783086 | 1.97 | 0.049 | .0007571 | .3077213 |
|  | JUDP | 1.181678 | .2206528 | 5.36 | 0.000 | .7492064 | 1.614149 |
|  | EEP | .006991 | .0117451 | 0.60 | 0.552 | -.0160289 | .030011 |
|  | EES | .058922 | .0108374 | 5.44 | 0.000 | .0376811 | .0801629 |
|  | EET | .002319 | .0014687 | 1.58 | 0.114 | -.0005596 | .0051975 |
|  | CONS | -1.128597 | .178961 | -6.31 | 0.000 | -1.479354 | -.7778403 |
| HLEB |  |  |  |  |  |  |  |
|  | lnGDP | 3.951827 | .1280041 | 30.87 | 0.000 | 3.700944 | 4.20271 |
|  | GINI | -.1467115 | .0120463 | -12.18 | 0.000 | -.1703218 | -.1231012 |
|  | **LSk3** | 3.290251 | .1228554 | 26.78 | 0.000 | 3.049459 | 3.531043 |
|  | BUDP | 2.606439 | .7618168 | 3.42 | 0.001 | 1.113306 | 4.099573 |
|  | CHRP | -.7879322 | .5357739 | -1.47 | 0.141 | -1.83803 | .2621654 |
|  | HINP | -2.103019 | .9550996 | -2.20 | 0.028 | -3.974979 | -.2310579 |
|  | ISLP | -.8358722 | .5060488 | -1.65 | 0.099 | -1.82771 | .1559652 |
|  | JUDP | -.5324797 | 1.434835 | -0.37 | 0.711 | -3.344704 | 2.279745 |
|  | EEP | .3591778 | .0754532 | 4.76 | 0.000 | .2112922 | .5070634 |
|  | EES | -.4929989 | .0696726 | -7.08 | 0.000 | -.6295547 | -.3564431 |
|  | EET | -.1165412 | .0091407 | -12.75 | 0.000 | -.1344566 | -.0986257 |
|  | _CONS | 15.62539 | 1.11782 | 13.98 | 0.000 | 13.43451 | 17.81628 |

**Table 15S. Impact of k at 3 on RELi & EDUi. R^2^ (ls) = 0.61, R^2^ (hleb) = 0.71.**

|  |  | Coef. | Std. Err. | z | P>z | [95% Conf. | Interval] |
| --- | --- | --- | --- | --- | --- | --- | --- |
| LS |  |  |  |  |  |  |  |
|  | lnGDP | .3238936 | .019798 | 16.36 | 0.000 | .2850904 | .3626969 |
|  | GINI | .0042548 | .0018635 | 2.28 | 0.022 | .0006024 | .0079073 |
|  | HLEB | .0794003 | .0030156 | 26.33 | 0.000 | .0734899 | .0853107 |
|  | BUDP | -.328452 | .118615 | -2.77 | 0.006 | -.560933 | -.095971 |
|  | CHRP | .31618 | .0825149 | 3.83 | 0.000 | .1544538 | .4779063 |
|  | HINP | .3627558 | .1503023 | 2.41 | 0.016 | .0681687 | .6573429 |
|  | ISLP | -.133557 | .0779015 | -1.71 | 0.086 | -.286241 | .0191271 |
|  | JUDP | 1.074936 | .2185669 | 4.92 | 0.000 | .6465531 | 1.50332 |
|  | GEP | -.0107661 | .0009074 | -11.87 | 0.000 | -.0125445 | -.0089877 |
|  | GES | .0012979 | .0008646 | 1.50 | 0.133 | -.0003967 | .0029925 |
|  | GET | -.0055205 | .0007607 | -7.26 | 0.000 | -.0070114 | -.0040296 |
|  | CONS | -1.515171 | .1974614 | -7.67 | 0.000 | -1.902188 | -1.128154 |
| HLEB |  |  |  |  |  |  |  |
|  | lnGDP | 1.1996 | .1297915 | 9.24 | 0.000 | .9452132 | 1.453987 |
|  | GINI | -.103814 | .0115231 | -9.01 | 0.000 | -.1263988 | -.0812292 |
|  | **LSk3** | 3.074856 | .1193987 | 25.75 | 0.000 | 2.840839 | 3.308873 |
|  | BUDP | 4.661562 | .7398052 | 6.30 | 0.000 | 3.211571 | 6.111554 |
|  | CHRP | -.4349665 | .5205591 | -0.84 | 0.403 | -1.455243 | .5853105 |
|  | HINP | -.9103092 | .9461335 | -0.96 | 0.336 | -2.764697 | .9440784 |
|  | ISLP | .858273 | .4895165 | 1.75 | 0.080 | -.1011617 | 1.817708 |
|  | JUDP | 1.247399 | 1.381351 | 0.90 | 0.367 | -1.459998 | 3.954797 |
|  | GEP | .061071 | .0057321 | 10.65 | 0.000 | .0498363 | .0723056 |
|  | GES | .0523711 | .0053178 | 9.85 | 0.000 | .0419484 | .0627938 |
|  | GET | .0513859 | .0047003 | 10.93 | 0.000 | .0421735 | .0605983 |
|  | CONS | 25.68262 | 1.129601 | 22.74 | 0.000 | 23.46865 | 27.8966 |

**Table 16S. Impact of k at 3 on RELs & EDUi. R^2^ (ls) = 0.61, R^2^ (hleb) = 0.70.**

|  |  | Coef. | Std. Err. | z | P>z | [95% Conf. | Interval] |
| --- | --- | --- | --- | --- | --- | --- | --- |
| LS |  |  |  |  |  |  |  |
|  | lnGDP | .0853911 | .0236919 | 3.60 | 0.000 | .0389559 | .1318264 |
|  | GINI | .0095608 | .0018917 | 5.05 | 0.000 | .0058532 | .0132684 |
|  | HLEB | .0794523 | .0028876 | 27.52 | 0.000 | .0737928 | .0851119 |
|  | BUDM | .0091804 | .0659307 | 0.14 | 0.889 | -.1200413 | .1384022 |
|  | CHRM | .3586446 | .0424834 | 8.44 | 0.000 | .2753787 | .4419104 |
|  | HINM | .1093776 | .1134525 | 0.96 | 0.335 | -.1129852 | .3317405 |
|  | ISLM | .1882505 | .0459922 | 4.09 | 0.000 | .0981075 | .2783935 |
|  | JUDM | .9823335 | .1574492 | 6.24 | 0.000 | .6737388 | 1.290928 |
|  | EEP | -.0013224 | .0117021 | -0.11 | 0.910 | -.0242581 | .0216132 |
|  | EES | .0617208 | .010823 | 5.70 | 0.000 | .0405081 | .0829334 |
|  | EET | .0023362 | .0014593 | 1.60 | 0.109 | -.0005241 | .0051964 |
|  | CONS | -1.123901 | .1698979 | -6.62 | 0.000 | -1.456895 | -.7909073 |
| HLEB |  |  |  |  |  |  |  |
|  | lnGDP | 3.938196 | .1290303 | 30.52 | 0.000 | 3.685301 | 4.191091 |
|  | GINI | -.1399812 | .0120372 | -11.63 | 0.000 | -.1635738 | -.1163887 |
|  | **LSk3** | 3.359172 | .1243181 | 27.02 | 0.000 | 3.115513 | 3.602831 |
|  | BUDM | .5439008 | .4292565 | 1.27 | 0.205 | -.2974265 | 1.385228 |
|  | CHRM | -1.126683 | .2805864 | -4.02 | 0.000 | -1.676622 | -.5767433 |
|  | HINM | .8162065 | .7389886 | 1.10 | 0.269 | -.6321846 | 2.264598 |
|  | ISLM | -1.096456 | .300011 | -3.65 | 0.000 | -1.684467 | -.5084454 |
|  | JUDM | -1.718765 | 1.034372 | -1.66 | 0.097 | -3.746096 | .3085659 |
|  | EEP | .4075315 | .0756969 | 5.38 | 0.000 | .2591683 | .5558947 |
|  | EES | -.5245225 | .0701262 | -7.48 | 0.000 | -.6619673 | -.3870777 |
|  | EET | -.1185498 | .0091489 | -12.96 | 0.000 | -.1364813 | -.1006182 |
|  | _CONS | 15.43894 | 1.068075 | 14.45 | 0.000 | 13.34555 | 17.53233 |

**Table 17S. Impact of k at 5 on RELs & EDUs. R^2^ (ls) = 0.61, R^2^ (hleb) = 0.71.**

|  |  | Coef. | Std. Err. | z | P>z | [95% Conf. | Interval] |
| --- | --- | --- | --- | --- | --- | --- | --- |
| LS |  |  |  |  |  |  |  |
|  | lnGDP | .3329741 | .0197234 | 16.88 | 0.000 | .2943169 | .3716312 |
|  | GINI | .0033016 | .0018373 | 1.80 | 0.072 | -.0002995 | .0069026 |
|  | HLEB | .0769898 | .0029868 | 25.78 | 0.000 | .0711358 | .0828438 |
|  | BUDM | -.0703423 | .0660414 | -1.07 | 0.287 | -.1997812 | .0590965 |
|  | CHRM | .4105821 | .0426668 | 9.62 | 0.000 | .3269567 | .4942075 |
|  | HINM | .2249024 | .1146301 | 1.96 | 0.050 | .0002316 | .4495732 |
|  | ISLM | .0511208 | .0455272 | 1.12 | 0.261 | -.0381108 | .1403524 |
|  | JUDM | .9518478 | .1557038 | 6.11 | 0.000 | .646674 | 1.257022 |
|  | GEP | -.0103074 | .0009057 | -11.38 | 0.000 | -.0120825 | -.0085323 |
|  | GES | .0012764 | .0008604 | 1.48 | 0.138 | -.00041 | .0029628 |
|  | GET | -.0054242 | .0007392 | -7.34 | 0.000 | -.006873 | -.0039755 |
|  | CONS | -1.589415 | .1890036 | -8.41 | 0.000 | -1.959855 | -1.218975 |
| HLEB |  |  |  |  |  |  |  |
|  | lnGDP | 1.269054 | .1308503 | 9.70 | 0.000 | 1.012592 | 1.525516 |
|  | GINI | -.1032527 | .0115172 | -8.97 | 0.000 | -.125826 | -.0806794 |
|  | **LSk5** | 2.941588 | .1191394 | 24.69 | 0.000 | 2.708078 | 3.175097 |
|  | BUDM | 1.313579 | .4196971 | 3.13 | 0.002 | .490988 | 2.136171 |
|  | CHRM | -1.639401 | .2751894 | -5.96 | 0.000 | -2.178762 | -1.10004 |
|  | HINM | -.229763 | .7309203 | -0.31 | 0.753 | -1.66234 | 1.202815 |
|  | ISLM | -.6079976 | .2898325 | -2.10 | 0.036 | -1.176059 | -.0399364 |
|  | JUDM | -.2700037 | .9993927 | -0.27 | 0.787 | -2.228777 | 1.68877 |
|  | GEP | .0584984 | .0057896 | 10.10 | 0.000 | .0471509 | .0698458 |
|  | GES | .054818 | .0053537 | 10.24 | 0.000 | .0443249 | .0653111 |
|  | GET | .0502907 | .0046262 | 10.87 | 0.000 | .0412235 | .0593579 |
|  | CONS | 27.11171 | 1.077003 | 25.17 | 0.000 | 25.00083 | 29.2226 |

**Table 18S. Impact of k at 5 on RELi & EDUi. R^2^ (ls) = 0.61, R^2^ (hleb) = 0.70.**

|  |  | Coef. | Std. Err. | z | P>z | [95% Conf. | Interval] |
| --- | --- | --- | --- | --- | --- | --- | --- |
| LS |  |  |  |  |  |  |  |
|  | lnGDP | .0951284 | .0238762 | 3.98 | 0.000 | .048332 | .1419248 |
|  | GINI | .0102232 | .0019125 | 5.35 | 0.000 | .0064748 | .0139715 |
|  | HLEB | .0769133 | .0029359 | 26.20 | 0.000 | .0711591 | .0826675 |
|  | BUDP | -.0845016 | .1182829 | -0.71 | 0.475 | -.3163319 | .1473287 |
|  | CHRP | .381246 | .0825473 | 4.62 | 0.000 | .2194563 | .5430357 |
|  | HINP | .5906802 | .1474417 | 4.01 | 0.000 | .3016998 | .8796606 |
|  | ISLP | .1533259 | .0783087 | 1.96 | 0.050 | -.0001564 | .3068081 |
|  | JUDP | 1.194017 | .2206575 | 5.41 | 0.000 | .7615363 | 1.626498 |
|  | EEP | .0084008 | .0117462 | 0.72 | 0.474 | -.0146214 | .031423 |
|  | EES | .0578248 | .0108381 | 5.34 | 0.000 | .0365824 | .0790672 |
|  | EET | .0019101 | .0014694 | 1.30 | 0.194 | -.0009699 | .0047902 |
|  | CONS | -1.085774 | .1790309 | -6.06 | 0.000 | -1.436669 | -.7348803 |
| HLEB |  |  |  |  |  |  |  |
|  | lsGDP | 4.076178 | .1277081 | 31.92 | 0.000 | 3.825875 | 4.326481 |
|  | GINI | -.1507182 | .0120645 | -12.49 | 0.000 | -.1743641 | -.1270723 |
|  | **LSk5** | 3.045015 | .1207465 | 25.22 | 0.000 | 2.808356 | 3.281674 |
|  | BUDP | 2.608268 | .7628426 | 3.42 | 0.001 | 1.113124 | 4.103412 |
|  | CHRP | -.695692 | .5364361 | -1.30 | 0.195 | -1.747087 | .3557034 |
|  | HINP | -2.045923 | .9565159 | -2.14 | 0.032 | -3.92066 | -.1711863 |
|  | ISLP | -.8639224 | .5067945 | -1.70 | 0.088 | -1.857221 | .1293765 |
|  | JUDP | -.1461999 | 1.436189 | -0.10 | 0.919 | -2.961078 | 2.668678 |
|  | EEP | .3751821 | .07553 | 4.97 | 0.000 | .2271461 | .5232181 |
|  | EES | -.4916584 | .0697712 | -7.05 | 0.000 | -.6284075 | -.3549093 |
|  | EET | -.1183733 | .0091526 | -12.93 | 0.000 | -.1363121 | -.1004344 |
|  | _CONS | 15.89115 | 1.119335 | 14.20 | 0.000 | 13.6973 | 18.08501 |

**Table 19S. Impact of k at 5 on RELi & EDUs. R^2^ (ls) = 0.61, R^2^ (hleb) = 0.72.**

|  |  | Coef. | Std. Err. | z | P>z | [95% Conf. | Interval] |
| --- | --- | --- | --- | --- | --- | --- | --- |
| LS |  |  |  |  |  |  |  |
|  | lnGDP | .3324999 | .0198208 | 16.78 | 0.000 | .2936518 | .371348 |
|  | GINI | .0039218 | .0018639 | 2.10 | 0.035 | .0002687 | .007575 |
|  | HLEB | .0764397 | .0030333 | 25.20 | 0.000 | .0704946 | .0823849 |
|  | BUDP | -.3138867 | .1186259 | -2.65 | 0.008 | -.5463892 | -.0813842 |
|  | CHRP | .3184434 | .0825153 | 3.86 | 0.000 | .1567164 | .4801703 |
|  | HINP | .3639733 | .1503023 | 2.42 | 0.015 | .0693862 | .6585605 |
|  | ISLP | -.1314339 | .0779018 | -1.69 | 0.092 | -.2841186 | .0212509 |
|  | JUDP | 1.092546 | .2185756 | 5.00 | 0.000 | .6641459 | 1.520946 |
|  | GEP | -.0106549 | .0009074 | -11.74 | 0.000 | -.0124335 | -.0088763 |
|  | GES | .001518 | .000865 | 1.76 | 0.079 | -.0001772 | .0032133 |
|  | GET | -.0053811 | .0007608 | -7.07 | 0.000 | -.0068723 | -.0038899 |
|  | CONS | -1.434194 | .1976644 | -7.26 | 0.000 | -1.821609 | -1.046779 |
| HLEB |  |  |  |  |  |  |  |
|  | lnGDP | 1.341758 | .1293615 | 10.37 | 0.000 | 1.088214 | 1.595302 |
|  | GINI | -.1088253 | .011541 | -9.43 | 0.000 | -.1314454 | -.0862053 |
|  | **LSk5** | 2.825455 | .1174974 | 24.05 | 0.000 | 2.595164 | 3.055745 |
|  | BUDP | 4.636692 | .7412362 | 6.26 | 0.000 | 3.183896 | 6.089488 |
|  | CHRP | -.3507237 | .5215224 | -0.67 | 0.501 | -1.372889 | .6714415 |
|  | HINP | -.9106449 | .9481307 | -0.96 | 0.337 | -2.768947 | .9476572 |
|  | ISLP | .7758852 | .4904219 | 1.58 | 0.114 | -.1853241 | 1.737094 |
|  | JUDP | 1.663832 | 1.38336 | 1.20 | 0.229 | -1.047503 | 4.375167 |
|  | GEP | .0589286 | .0057389 | 10.27 | 0.000 | .0476806 | .0701766 |
|  | GES | .0540417 | .0053262 | 10.15 | 0.000 | .0436025 | .064481 |
|  | GET | .0502688 | .0047081 | 10.68 | 0.000 | .0410411 | .0594966 |
|  | CONS | 26.0381 | 1.131203 | 23.02 | 0.000 | 23.82098 | 28.25521 |

**Table 20S. Impact of k at 5 on RELs & EDUi. R^2^ (ls) = 0.61, R^2^ (hleb) = 0.70.**

|  |  | Coef. | Std. Err. | z | P>z | [95% Conf. | Interval] |
| --- | --- | --- | --- | --- | --- | --- | --- |
| LS |  |  |  |  |  |  |  |
|  | lnGDP | .1014627 | .0237657 | 4.27 | 0.000 | .0548827 | .1480426 |
|  | GINI | .0091728 | .0018922 | 4.85 | 0.000 | .0054641 | .0128814 |
|  | HLEB | .0766758 | .0029056 | 26.39 | 0.000 | .0709809 | .0823708 |
|  | BUDM | .0112941 | .0659312 | 0.17 | 0.864 | -.1179286 | .1405168 |
|  | CHRM | .3590116 | .0424834 | 8.45 | 0.000 | .2757457 | .4422775 |
|  | HINM | .1141554 | .1134539 | 1.01 | 0.314 | -.10821 | .3365209 |
|  | ISLM | .1866799 | .0459925 | 4.06 | 0.000 | .0965361 | .2768236 |
|  | JUDM | .9881222 | .1574506 | 6.28 | 0.000 | .6795247 | 1.29672 |
|  | EEP | .000188 | .0117034 | 0.02 | 0.987 | -.0227503 | .0231263 |
|  | EES | .0605351 | .0108239 | 5.59 | 0.000 | .0393207 | .0817495 |
|  | EET | .0019138 | .0014602 | 1.31 | 0.190 | -.0009481 | .0047757 |
|  | CONS | -1.081181 | .1699708 | -6.36 | 0.000 | -1.414317 | -.7480443 |
| HLEB |  |  |  |  |  |  |  |
|  | lnGDP | 4.067815 | .1287234 | 31.60 | 0.000 | 3.815522 | 4.320108 |
|  | GINI | -.1439454 | .0120537 | -11.94 | 0.000 | -.1675702 | -.1203206 |
|  | **LSk5** | 3.107694 | .1222214 | 25.43 | 0.000 | 2.868145 | 3.347244 |
|  | BUDM | .5589846 | .4298018 | 1.30 | 0.193 | -.2834114 | 1.401381 |
|  | CHRM | -1.047817 | .2809042 | -3.73 | 0.000 | -1.59838 | -.4972552 |
|  | HINM | .7847496 | .740087 | 1.06 | 0.289 | -.6657943 | 2.235293 |
|  | ISLM | -1.105536 | .3004998 | -3.68 | 0.000 | -1.694505 | -.5165671 |
|  | JUDM | -1.425057 | 1.035299 | -1.38 | 0.169 | -3.454205 | .6040911 |
|  | EEP | .4235744 | .0757727 | 5.59 | 0.000 | .2750627 | .5720861 |
|  | EES | -.5231152 | .0702243 | -7.45 | 0.000 | -.6607523 | -.3854781 |
|  | EET | -.1206061 | .00916 | -13.17 | 0.000 | -.1385593 | -.1026529 |
|  | _CONS | 15.6886 | 1.0695 | 14.67 | 0.000 | 13.59242 | 17.78478 |

**Table 21S. Impact of GDP at t-1 on RELs & EDUs. R^2^ (ls) = 0.62, R^2^ (hleb) = 0.71.**

|  |  | Coef. | Std. Err. | z | P>z | [95% Conf. | Interval] |
| --- | --- | --- | --- | --- | --- | --- | --- |
| LS | lnGDP | .5407206 | .2100597 | 2.57 | 0.010 | .1290111 | .9524301 |
|  | **lnGDPt-1** | -.1970598 | .2064316 | -0.95 | 0.340 | -.6016583 | .2075387 |
|  | GINI | .0031051 | .0018638 | 1.67 | 0.096 | -.0005478 | .0067581 |
|  | HLEB | .0763038 | .0030297 | 25.19 | 0.000 | .0703656 | .0822419 |
|  | BUDM | -.0553579 | .0665726 | -0.83 | 0.406 | -.1858378 | .075122 |
|  | CHRM | .4234021 | .043013 | 9.84 | 0.000 | .3390981 | .5077061 |
|  | HINM | .2431777 | .1155779 | 2.10 | 0.035 | .0166491 | .4697063 |
|  | ISLM | .0725552 | .0461002 | 1.57 | 0.116 | -.0177995 | .1629099 |
|  | JUDM | .9820633 | .1569508 | 6.26 | 0.000 | .6744453 | 1.289681 |
|  | GEP | -.0103582 | .0009246 | -11.20 | 0.000 | -.0121704 | -.008546 |
|  | GES | .0011535 | .0008745 | 1.32 | 0.187 | -.0005605 | .0028675 |
|  | GET | -.0053459 | .0007435 | -7.19 | 0.000 | -.0068031 | -.0038886 |
|  | CONS | -1.66656 | .1954325 | -8.53 | 0.000 | -2.049601 | -1.28352 |
| HLEB | lnGDP | 4.278303 | 1.346675 | 3.18 | 0.001 | 1.638868 | 6.917738 |
|  | **lnGDPt-1** | -3.066047 | 1.324835 | -2.31 | 0.021 | -5.662675 | -.4694181 |
|  | GINI | -.1018232 | .0117863 | -8.64 | 0.000 | -.1249239 | -.0787226 |
|  | LSk4 | 3.051615 | .1245934 | 24.49 | 0.000 | 2.807416 | 3.295813 |
|  | BUDM | 1.226754 | .4269569 | 2.87 | 0.004 | .3899336 | 2.063574 |
|  | CHRM | -1.626215 | .2805468 | -5.80 | 0.000 | -2.176077 | -1.076354 |
|  | HINM | -.2888312 | .7437017 | -0.39 | 0.698 | -1.74646 | 1.168797 |
|  | ISLM | -.6079177 | .2961823 | -2.05 | 0.040 | -1.188424 | -.0274112 |
|  | JUDM | -.303382 | 1.017508 | -0.30 | 0.766 | -2.297661 | 1.690897 |
|  | GEP | .0584683 | .005974 | 9.79 | 0.000 | .0467595 | .0701771 |
|  | GES | .0533094 | .005497 | 9.70 | 0.000 | .0425355 | .0640832 |
|  | GET | .0502258 | .0046959 | 10.70 | 0.000 | .041022 | .0594295 |
|  | CONS | 27.04513 | 1.129891 | 23.94 | 0.000 | 24.83058 | 29.25967 |

**Table 22S. Impact of GDP at t-1 on RELi & EDUi. R^2^ (ls) = 0.62, R^2^ (hleb) = 0.70.**

|  |  | Coef. | Std. Err. | z | P>z | [95% Conf. | Interval] |
| --- | --- | --- | --- | --- | --- | --- | --- |
| LS | lnGDP | .575183 | .2097955 | 2.74 | 0.006 | .1639915 | .9863746 |
|  | **lnGDPt-1** | -.4706775 | .2078843 | -2.26 | 0.024 | -.8781233 | -.0632318 |
|  | GINI | .0106367 | .0019459 | 5.47 | 0.000 | .0068228 | .0144506 |
|  | HLEB | .0759317 | .0029706 | 25.56 | 0.000 | .0701095 | .0817539 |
|  | BUDP | -.0708462 | .1191712 | -0.59 | 0.552 | -.3044175 | .162725 |
|  | CHRP | .3974917 | .0833281 | 4.77 | 0.000 | .2341717 | .5608117 |
|  | HINP | .6201217 | .1485219 | 4.18 | 0.000 | .3290242 | .9112192 |
|  | ISLP | .188969 | .0794132 | 2.38 | 0.017 | .0333221 | .3446159 |
|  | JUDP | 1.227078 | .2223947 | 5.52 | 0.000 | .7911926 | 1.662964 |
|  | EEP | .0120241 | .0116552 | 1.03 | 0.302 | -.0108197 | .034868 |
|  | EES | .056511 | .0107812 | 5.24 | 0.000 | .0353802 | .0776417 |
|  | EET | .0017772 | .0014906 | 1.19 | 0.233 | -.0011444 | .0046988 |
|  | CONS | -1.192096 | .1828035 | -6.52 | 0.000 | -1.550384 | -.8338078 |
| HLEB | lnGDP | 6.216408 | 1.365317 | 4.55 | 0.000 | 3.540437 | 8.89238 |
|  | **lnGDPt-1** | -2.266856 | 1.358515 | -1.67 | 0.095 | -4.929497 | .3957844 |
|  | GINI | -.1490242 | .0124226 | -12.00 | 0.000 | -.173372 | -.1246763 |
|  | LSK4 | 3.156955 | .1265546 | 24.95 | 0.000 | 2.908913 | 3.404997 |
|  | BUDP | 2.555633 | .7772065 | 3.29 | 0.001 | 1.032336 | 4.07893 |
|  | CHRP | -.6818534 | .5478313 | -1.24 | 0.213 | -1.755583 | .3918762 |
|  | HINP | -2.140584 | .9745899 | -2.20 | 0.028 | -4.050745 | -.2304229 |
|  | ISLP | -.8589574 | .5198335 | -1.65 | 0.098 | -1.877812 | .1598976 |
|  | JUDP | -.1351902 | 1.464683 | -0.09 | 0.926 | -3.005916 | 2.735536 |
|  | EEP | .3495247 | .0758313 | 4.61 | 0.000 | .200898 | .4981513 |
|  | EES | -.4626825 | .0702437 | -6.59 | 0.000 | -.6003577 | -.3250073 |
|  | EET | -.1161599 | .0094011 | -12.36 | 0.000 | -.1345857 | -.0977341 |
|  | CONS | 16.31122 | 1.154947 | 14.12 | 0.000 | 14.04756 | 18.57487 |

**Table 23S. Impact of GDP at t-1 on RELi & EDUs. R^2^ (ls) = 0.62, R^2^ (hleb) = 0.71.**

|  |  | Coef. | Std. Err. | z | P>z | [95% Conf. | Interval] |
| --- | --- | --- | --- | --- | --- | --- | --- |
| LS | lnGDP | .4891712 | .2117627 | 2.31 | 0.021 | .074124 | .9042183 |
|  | **lnGDPt-1** | -.1472943 | .2081082 | -0.71 | 0.479 | -.5551789 | .2605902 |
|  | GINI | .0037281 | .0018933 | 1.97 | 0.049 | .0000172 | .0074389 |
|  | HLEB | .0760507 | .0030787 | 24.70 | 0.000 | .0700165 | .0820849 |
|  | BUDP | -.3179664 | .119696 | -2.66 | 0.008 | -.5525662 | -.0833666 |
|  | CHRP | .321902 | .0834242 | 3.86 | 0.000 | .1583936 | .4854104 |
|  | HINP | .3737835 | .1517126 | 2.46 | 0.014 | .0764322 | .6711348 |
|  | ISLP | -.1185961 | .0790342 | -1.50 | 0.133 | -.2735004 | .0363081 |
|  | JUDP | 1.11732 | .2207433 | 5.06 | 0.000 | .6846714 | 1.549969 |
|  | GEP | -.0106774 | .0009272 | -11.52 | 0.000 | -.0124946 | -.0088602 |
|  | GES | .0013883 | .00088 | 1.58 | 0.115 | -.0003365 | .003113 |
|  | GET | -.005326 | .0007664 | -6.95 | 0.000 | -.0068281 | -.0038239 |
|  | CONS | -1.508641 | .2050468 | -7.36 | 0.000 | -1.910526 | -1.106757 |
| HLEB | lnGDP | 4.384534 | 1.341051 | 3.27 | 0.001 | 1.756122 | 7.012947 |
|  | **lnGDPt-1** | -3.100088 | 1.319731 | -2.35 | 0.019 | -5.686713 | -.5134639 |
|  | GINI | -.1070685 | .011816 | -9.06 | 0.000 | -.1302275 | -.0839096 |
|  | LSk4 | 2.938117 | .1226985 | 23.95 | 0.000 | 2.697633 | 3.178602 |
|  | BUDP | 4.576603 | .7541315 | 6.07 | 0.000 | 3.098532 | 6.054673 |
|  | CHRP | -.3138815 | .5315827 | -0.59 | 0.555 | -1.355764 | .7280015 |
|  | HINP | -.9523428 | .9647672 | -0.99 | 0.324 | -2.843252 | .9385662 |
|  | ISLP | .8105443 | .5015774 | 1.62 | 0.106 | -.1725293 | 1.793618 |
|  | JUDP | 1.627981 | 1.409113 | 1.16 | 0.248 | -1.133831 | 4.389792 |
|  | GEP | .0588489 | .0059205 | 9.94 | 0.000 | .0472448 | .0704529 |
|  | GES | .0525567 | .0054689 | 9.61 | 0.000 | .0418379 | .0632755 |
|  | GET | .0503114 | .0047818 | 10.52 | 0.000 | .0409391 | .0596836 |
|  | CONS | 25.92204 | 1.189038 | 21.80 | 0.000 | 23.59157 | 28.25251 |

**Table 24S. Impact of GDP at t-1 on RELs & EDUi. R^2^ (ls) = 0.63, R^2^ (hleb) = 0.70.**

|  |  | Coef. | Std. Err. | z | P>z | [95% Conf. | Interval] |
| --- | --- | --- | --- | --- | --- | --- | --- |
| LS | lnGDP | .5886843 | .2083172 | 2.83 | 0.005 | .1803901 | .9969785 |
|  | **lnGDPt-1** | -.4768066 | .2063852 | -2.31 | 0.021 | -.8813141 | -.072299 |
|  | GINI | .0095519 | .0019243 | 4.96 | 0.000 | .0057804 | .0133234 |
|  | HLEB | .0754674 | .0029395 | 25.67 | 0.000 | .0697061 | .0812286 |
|  | BUDM | .027781 | .0663335 | 0.42 | 0.675 | -.1022303 | .1577922 |
|  | CHRM | .3702695 | .042748 | 8.66 | 0.000 | .2864849 | .4540541 |
|  | HINM | .1352606 | .1141153 | 1.19 | 0.236 | -.0884014 | .3589226 |
|  | ISLM | .2156892 | .0465473 | 4.63 | 0.000 | .1244581 | .3069203 |
|  | JUDM | 1.016413 | .1584133 | 6.42 | 0.000 | .7059283 | 1.326897 |
|  | EEP | .0033253 | .0116054 | 0.29 | 0.774 | -.0194208 | .0260714 |
|  | EES | .0596848 | .0107634 | 5.55 | 0.000 | .0385889 | .0807807 |
|  | EET | .0017297 | .0014805 | 1.17 | 0.243 | -.001172 | .0046314 |
|  | CONS | -1.17721 | .173193 | -6.80 | 0.000 | -1.516662 | -.837758 |
| HLEB | lnGDP | 6.415013 | 1.367528 | 4.69 | 0.000 | 3.734709 | 9.095318 |
|  | **lnGDPt-1** | -2.472273 | 1.36067 | -1.82 | 0.069 | -5.139138 | .1945922 |
|  | GINI | -.1422464 | .0124107 | -11.46 | 0.000 | -.166571 | -.1179218 |
|  | LSk4 | 3.217113 | .128245 | 25.09 | 0.000 | 2.965758 | 3.468469 |
|  | BUDM | .4848041 | .4375254 | 1.11 | 0.268 | -.37273 | 1.342338 |
|  | CHRM | -1.046448 | .2864609 | -3.65 | 0.000 | -1.607902 | -.4849954 |
|  | HINM | .7297024 | .7531721 | 0.97 | 0.333 | -.7464878 | 2.205893 |
|  | ISLM | -1.119149 | .3079643 | -3.63 | 0.000 | -1.722748 | -.5155501 |
|  | JUDM | -1.415469 | 1.054867 | -1.34 | 0.180 | -3.482969 | .6520319 |
|  | EEP | .3985553 | .0760551 | 5.24 | 0.000 | .2494901 | .5476205 |
|  | EES | -.4950928 | .0707201 | -7.00 | 0.000 | -.6337017 | -.3564839 |
|  | EET | -.1182389 | .0094097 | -12.57 | 0.000 | -.1366815 | -.0997962 |
|  | CONS | 16.11188 | 1.101607 | 14.63 | 0.000 | 13.95277 | 18.27098 |
